# Early treatment with a combination of two potent neutralizing antibodies improves clinical outcomes and reduces virus replication and lung inflammation in SARS-CoV-2 infected macaques

**DOI:** 10.1371/journal.ppat.1009688

**Published:** 2021-07-06

**Authors:** Koen K. A. Van Rompay, Katherine J. Olstad, Rebecca L. Sammak, Joseph Dutra, Jennifer K. Watanabe, Jodie L. Usachenko, Ramya Immareddy, Anil Verma, Yashavanth Shaan Lakshmanappa, Brian A. Schmidt, Jamin W. Roh, Sonny R. Elizaldi, A. Mark Allen, Frauke Muecksch, Julio C. C. Lorenzi, Sarah Lockwood, Rachel E. Pollard, JoAnn L. Yee, Peter B. Nham, Amir Ardeshir, Jesse D. Deere, Jean Patterson, Que Dang, Theodora Hatziioannou, Paul D. Bieniasz, Smita S. Iyer, Dennis J. Hartigan-O’Connor, Michel C. Nussenzweig, J. Rachel Reader

**Affiliations:** 1 California National Primate Research Center, University of California, Davis, United States of America; 2 Department of Pathology, Microbiology and Immunology, University of California, Davis, California, United States of America; 3 Department of Medical Microbiology and Immunology, School of Medicine, University of California, Davis, California, United States of America; 4 Center for Immunology and Infectious Diseases, University of California, Davis, California, United States of America; 5 Laboratory of Retrovirology, The Rockefeller University, New York, New York, United States of America; 6 Laboratory of Molecular Immunology, The Rockefeller University, New York, New York, United States of America; 7 School of Veterinary Medicine, University of California, Davis, California, United States of America; 8 Translational Research Section, Virology Branch, DMID/NIAID/NIH, Rockville, Maryland, United States of America; 9 Preclinical Research and Development Branch, Vaccine Research Program, DAIDS/NIAID/NIH, Rockville, Maryland, United States of America; 10 Howard Hughes Medical Institute, The Rockefeller University, New York, New York, United States of America; Chang Gung University, TAIWAN

## Abstract

There is an urgent need for effective therapeutic interventions against SARS-CoV-2, including new variants that continue to arise. Neutralizing monoclonal antibodies have shown promise in clinical studies. We investigated the therapeutic efficacy of a combination of two potent monoclonal antibodies, C135-LS and C144-LS that carry half-life extension mutations, in the rhesus macaque model of COVID-19. Twelve young adult macaques (three groups of four animals) were inoculated intranasally and intra-tracheally with a high dose of SARS-CoV-2 and 24 hours later, treated intravenously with a high (40 mg/kg) or low (12 mg/kg) dose of the C135-LS and C144-LS antibody combination, or a control monoclonal antibody. Animals were monitored for 7 days. Compared to the control animals, animals treated with either dose of the anti-SARS-CoV-2 antibodies showed similarly improved clinical scores, lower levels of virus replication in upper and lower respiratory tract, and significantly reduced interstitial pneumonia, as measured by comprehensive lung histology. In conclusion, this study provides proof-of-concept in support of further clinical development of these monoclonal antibodies against COVID-19 during early infection.

## Author summary

Monoclonal antibodies that neutralize SARS-CoV-2 have shown promise in treating recently infected individuals who are at high risk of progression to severe COVID-19 disease. Although several monoclonal antibodies are currently being used in the clinic, there is an ongoing need to develop additional antibodies. The ideal monoclonal antibodies, or combinations, should be potent and durable, and maintain activity against emerging viral variants. In this study, we tested a combination of two potent monoclonal antibodies, C135-LS and C-144-LS, engineered to have long half-lives, in the macaque model of SARS-CoV-2 infection. Animals treated early after infection fared better than placebo-treated controls, manifesting fewer clinical signs, less virus replication in the respiratory tract, and reduced lung inflammation. These promising data support clinical testing of these monoclonal antibodies in humans and further development of similar antibody-based prophylactic and therapeutic strategies.

## Introduction

In late 2019, a newly identified coronavirus, SARS-CoV-2, associated with a severe acute respiratory syndrome (SARS), also called COVID-19, began to spread rapidly from Wuhan, China across the globe [1].

While several effective vaccines are currently in use, there is also an urgent need for therapies for infected individuals, especially those at risk of developing serious disease. Currently, remdesivir is the only small molecule antiviral drug that received approval by the Food and Drug Administration to treat infection, but the benefits are moderate and most pronounced when treatment is started early during the course of infection [2]; in addition, challenges associated with production scale-up and cost still limit widespread global application. Thus, there is an urgent need for additional interventions.

Although there was initial interest in using convalescent plasma (CP) for therapy, including Emergency Use Authorization, the preliminary data indicate that its benefits are minimal, and then only at high CP antibody titers given during the early stages of disease [3–10]. The expanded use of CP is hampered in part due to the high level of individual variability in composition of CP, and the observation that most convalescent individuals don’t develop high titers of neutralizing antibodies [11].

An alternative approach for antibody-based therapies is the use of potent neutralizing monoclonal antibodies (mAbs). Several groups have isolated potent neutralizing mAbs and demonstrated their efficacy in animal models when administered prophylactically or therapeutically [11–17]. In addition, bamlanivimab, and the combination of casirivimab/imdevimab have demonstrated efficacy preventing mild COVID-19 infection from progressing into severe disease, particularly when given early in infection [18,19]. These mAbs have received emergency use authorization for treatment of high-risk ambulatory patients with mild or moderate COVID-19.

Despite the promise of this first group of mAbs there is an urgent need for the development of additional antibodies that have greater durability and can be used at low doses to meet national and international demands. In addition, the emergence of new viral variants, some of which have reduced susceptibility to the first-generation mAbs [20], also emphasizes the need for the development of mAbs with broad-spectrum activity.

SARS-CoV-2 infection of nonhuman primates is a relevant animal model to study pathogenesis because it recapitulates many of the key features of the human disease including high levels of virus replication, immunological responses to infection, and the development of interstitial pneumonia [21,22]. The rhesus macaque model has been used to demonstrate the efficacy of several prophylactic and therapeutic interventions [15–17,23–25].

Here we report on the use of the rhesus macaque model to investigate the therapeutic efficacy of a combination of two mAbs obtained from convalescent individuals, C135-LS and C144-LS. The two antibodies bind to complementary sites on the SARS-CoV-2 receptor-binding domain (RBD), and display 50% inhibitory concentrations (IC_50_) of ~ 3 ng/ml in a real-virus neutralization assay [11]. The combination of the two antibodies was found to elevate the genetic barrier to the acquisition of resistance by a rVSV/SARS-CoV-2 chimeric virus and be fully active *in vitro* against pseudotype viruses that carry RBD mutations corresponding to several different newly emerging variants [20,26]. Both antibodies were produced as human IgG1s with an M428L/N434S (LS) mutation to prolong their half-life [27]. As has been demonstrated for other viral infections, including ZIKV [28] and HIV-1 [29], combining two 2 potent mAbs targeting complementary epitopes reduces the risk of emergence of immune escape mutants [26].

We find that administration of the combination of C135-LS and C144-LS, one day after SARS-CoV-2 infection, had significant therapeutic efficacy as defined by improved clinical outcome, reduced virus replication in upper and lower respiratory tract, and reduced lung inflammation. These results support the further clinical development of these mAbs and highlight the value of the macaque model to test therapeutic strategies against COVID-19.

## Results

### Summary of experimental design to test therapeutic efficacy of monoclonal antibodies

**Fig 1:**
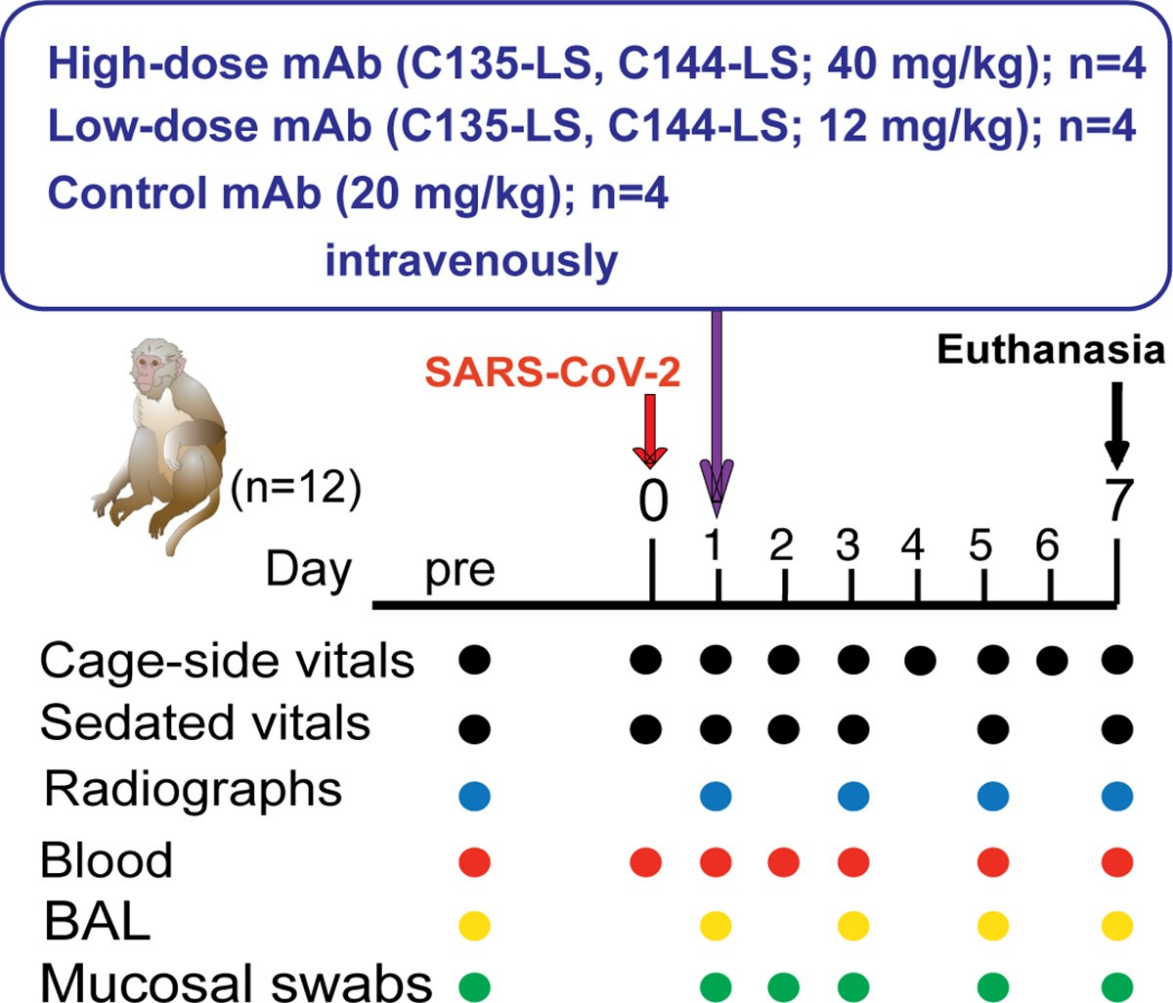
Experimental design. Three groups of adult rhesus macaques were inoculated on day 0 with SARS-CoV-2 by both intratracheal and intranasal routes. On day 1, anti-SARS-CoV-2- mAbs C135-LS and C144-LS (at 2 different dose regimens) or an unrelated control mAb were administered intravenously. Animals were monitored closely for clinical signs (both cage-side and sedated observations) with regular collection of radiographs and samples to monitor infection. On day 7, animals were euthanized for detailed tissue collection.

Three groups of 4 young adult macaques were inoculated with a high dose (2.5 x10^6^ PFU) of a Washington isolate of SARS-CoV-2 by the intratracheal and intranasal routes. After ~24 hours, animals were treated with either a high (40 mg/kg) or low dose (12 mg/kg) of a 1:1 mixture of C135-LS and C144-LS (CoV-2-mAb), or with a control mAb. Animals were monitored closely and were euthanized for tissue collection on day 7 (Fig 1).

### High neutralizing activity in serum after mAb administration

**Fig 2:**
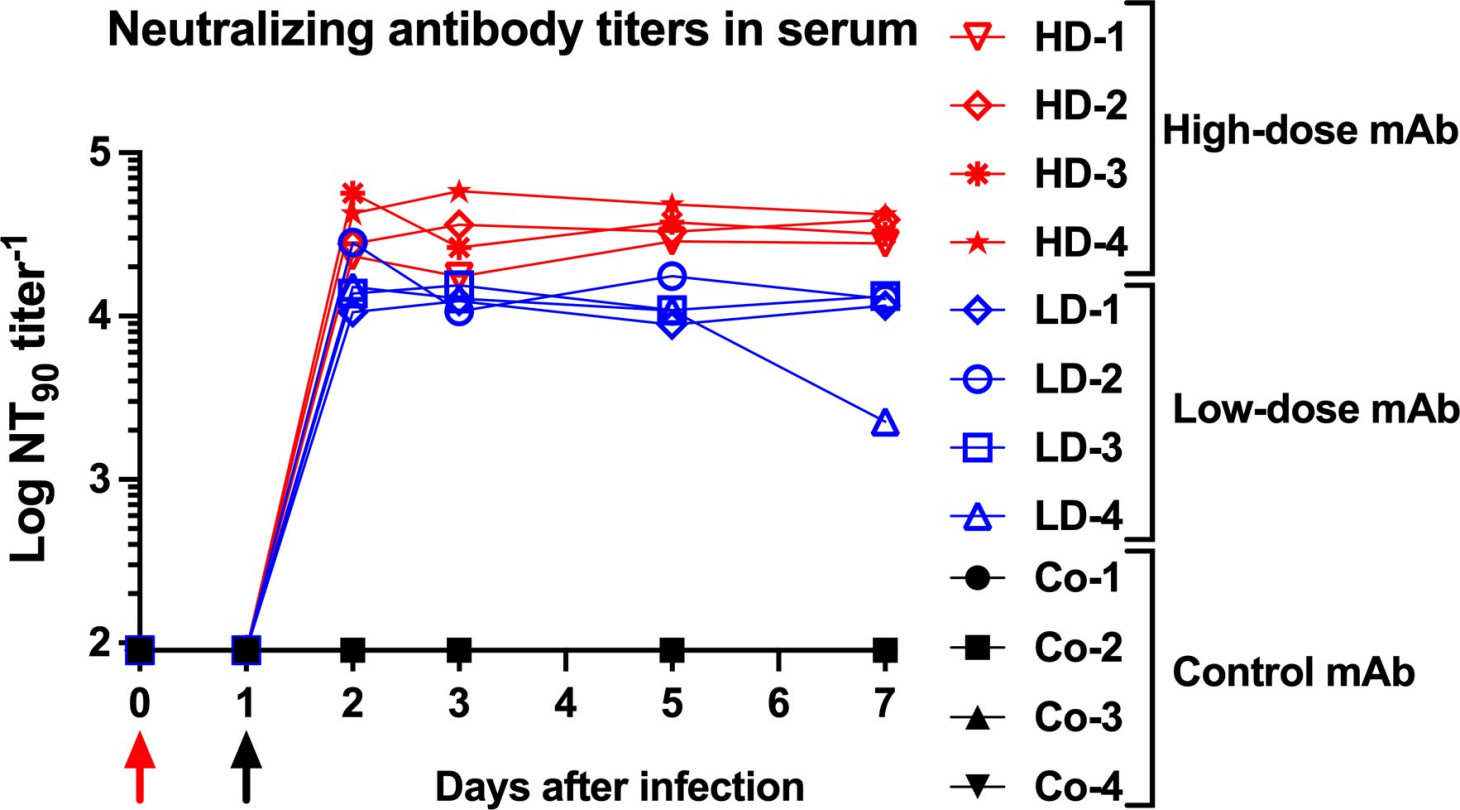
Neutralizing activity in serum of macaques after administration of CoV-2 monoclonal antibodies. Animals were inoculated on day 0 (red arrow) and administered antibodies on day 1 (black arrow). Neutralizing activity was measured in serum samples using a RVPN assay, with estimation of the titer to get 90% inhibition. Samples with undetectable titers are presented at the limit of detection (1:90).

After infusion of the antibodies on day 1, the CoV-2-mAb-treated animals had persistently high neutralizing activity in serum samples. After 2–3 days, neutralizing antibody titers reached NT_90_ values of 17,797 and 43,609 for the low- and high-dose mAb groups, respectively. Neutralizing activity remained high throughout the 7-day experiment with NT_90_ titers remaining ≥ 2,257 and ≥ 17,522, for the low- and high-dose CoV-2-mAb groups, respectively (Fig 2 and S1 Table). The concentrations of CoV-2-mAb in serum, estimated based on the neutralizing titers, reflected the ~3-fold difference in doses between the low- and high-dose CoV-2-mAb groups (S1 Table).

### Improved clinical outcome following CoV2-mAb treatment

**Fig 3:**
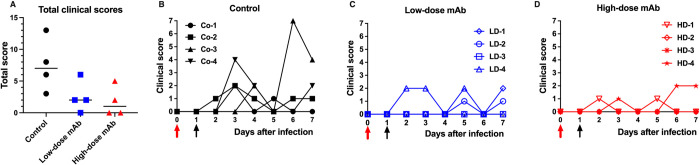
Improvement of clinical scores by CoV2-mAb treatment. Red and black arrows indicate time of virus inoculation and mAb administration on days 0 and 1, respectively. (A) For each animal, the total of clinical scores based on cage-side observations over the 7-day period were calculated; comparison of the 3 groups revealed therapeutic benefits of the mAb treatment (comparison of the 3 groups, p = 0.05, Kruskal-Wallis; comparison of control group versus combined mAb groups, p = 0.016, Mann-Whitney). (B through C) Daily clinical scores (based on cage-side observations) for each animal of the 3 study groups; the maximum daily score possible is 22.

Animals were scored daily for several clinical parameters by cage-side observation. Overall, clinical signs were absent or mild-to-moderate (daily scores ≤ 7 out of a maximum of 22) and consisted mostly of nasal discharge and occasional sneezing and coughing. The highest daily score of 7 was recorded for control animal Co-3. When the sums of the daily scores from day 0 to 7 were tabulated for each animal, control animals had higher scores than the 2 groups of CoV2-mAb treated groups, indicating a clinical therapeutic benefit from CoV-2-mAb treatment (Fig 3; p = 0.016, Mann-Whitney).

All animals had stable weights throughout the observation period, consistent with an adequate appetite. Four animals had at least one recording of elevated rectal temperature (> 103° F), particularly at day 1 (i.e., prior to mAb administration; S1 Fig), which was treated with a dose of ketoprofen. Three of the control animals, but none of the CoV-2-mAb treated animals, developed tachypnea (respiratory rate > 55/minute; S1 Fig) between days 2 and 7. None of the animals developed low oxygen saturation levels (Sp02 < 95%).

**Fig 4:**
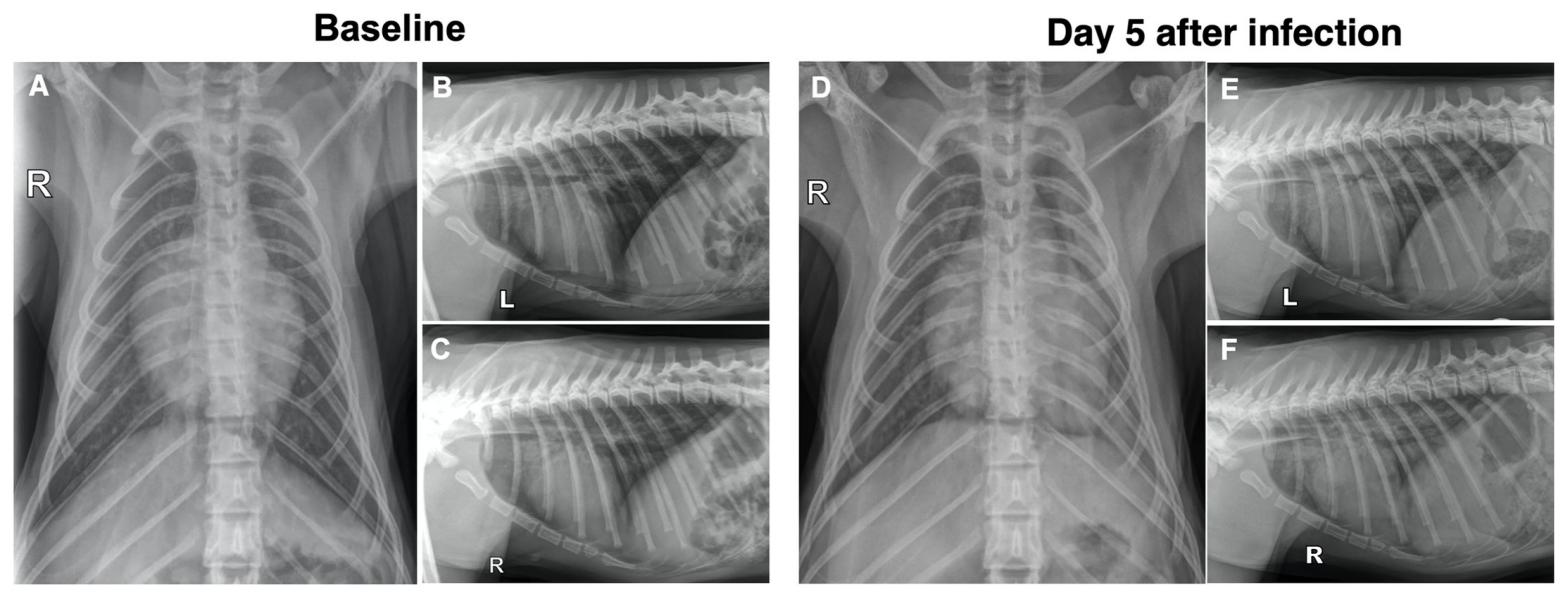
Radiologic scoring of pulmonary infiltration. (A to C) Ventrodorsal, left laterally recumbent and right laterally recumbent radiographs of control animal Co-3 on the day prior to inoculation showing no evidence of significant pulmonary pathology. (D to F) Ventrodorsal, left laterally recumbent and right laterally recumbent radiographs of animal Co-3 on day 5 after SARS-CoV-2 inoculation showing grade 1 infiltrates in the right caudal, cranial segment of the left cranial and caudal segment of the left cranial lung lobes. Grade 2 infiltrates are seen in the left caudal lung lobes yielding a total radiographic score of 5. R and L indicate right and left side, respectively.

Control animal Co-3 had evidence of pulmonary infiltrates on radiographs (Fig 4; daily total radiology scores were ≥ 4 from day 3 to day 7). All other animals in the study had normal to low radiology scores (daily total score ≤ 2) throughout the observation period, making group differences not significant (S2 Table).

### Increased inflammation and T cell proliferation following SARS-CoV-2 infection

Following infection, most animals had a transient increase of several cytokines and chemokines, including interferon (IFN)-alpha and MCP-1 (S2 Fig) peaking on day 1 (i.e., just before mAb administration), and a transient increase in C-reactive protein, ALT and AST, peaking on day 2 (S3 Fig). As we have shown previously [21], these markers of inflammation declined rapidly back to baseline levels even in the control animals, with no discernible differences from the CoV-2-mAb-treated groups. Other cytokines (including IFN-beta and IFN-gamma), chemokines and serum chemistries did not show consistent changes (S2 and S3 Figs).

Based on the aforementioned increase in inflammatory markers, we performed phenotypic analysis on peripheral blood mononuclear cells (PBMCs) to determine if a corresponding increase in proliferating T cells was observed after infection. The data showed a significant increase in circulating Ki-67^+^ CD4^+^ T cells, specificially the CXCR3 subset, denoting induction of T_h_1 CD4^+^ T cells (S4A and S4B Fig). Similar increases in Ki-67^+^ CD8^+^ T cell frequencies were also observed (S4C Fig). Induction of Ki-67^+^ T cells, independent of treatment group, indicated that antigen loads were not limiting during T cell priming.

### CoV-2 monoclonal antibody treatment reduces virus replication in upper and lower respiratory tract

Nasal swabs, oropharyngeal swabs, and broncho-alveolar lavages (BAL) were collected regularly for viral load analysis. In addition, at the time of euthanasia, small specimens of 6 lung lobes were collected and processed. Samples were tested by RT-qPCR for total viral RNA (vRNA, N target), genomic viral RNA (gRNA, ORF1a target), subgenomic viral RNA (sgRNA, leader/N target), and cellular mRNA of the housekeeping gene PPIA. In general, and as expected, the relative ratios of the 3 types of viral RNA’s were quite consistent in the samples (vRNA>gRNA>sgRNA). Since sgRNA is the best measure of active virus replication, we focused on sgRNA; data on vRNA and gRNA are presented in the supplement (S5 and S6 Figs).

**Fig 5:**
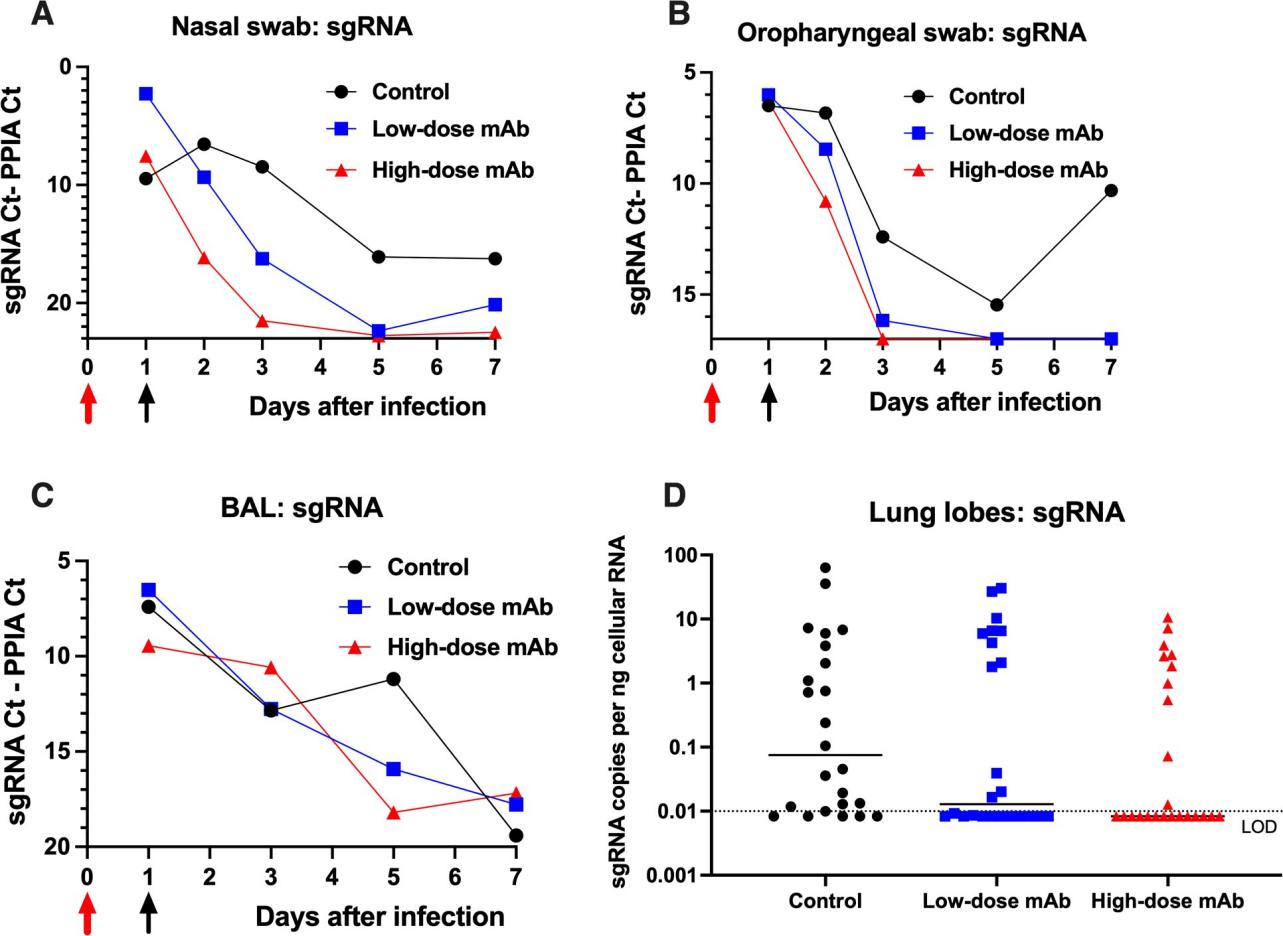
Reduced virus replication in upper and lower respiratory tract by anti-SARS-CoV-2 antibody treatment. (A) Time course of median viral sgRNA copies in nasal swabs. (B) Time course of median viral sgRNA copies in oropharyngeal swabs. Using a weighted average analysis relative to day 1 (for details, see S7 Fig), treatment with anti-SARS-CoV-2 mAbs resulted in more rapid decline of sgRNA relative to the control group (ANOVA, p = 0.016 and 0.006 for nasal and oropharyngeal swabs, respectively). (C) Time course of viral sgRNA in BAL cell pellets. In comparison to the control group, the 2 antibody-treated groups had a more rapid decline in sgRNA; however, due to high intra-group individual variability, this was statistically not significantly different. The largest difference was on day 5 (combined mAb-treated group combined versus controls, one-sided t-test on log-transformed dCt values, p = 0.056). Red and black arrows on panels A-C indicate time of virus inoculation and monoclonal antibody administration on days 0 and 1, respectively. Viral subgenomic (sg) RNA was measured by RT-qPCR and expressed relative to cellular mRNA of the housekeeping gene PPIA, as indicator of the cellular content in the sample tested. Values are expressed by difference in CT values to mRNA (graphs A-C), with a higher difference indicating a reduction in viral level. In graphs A to C, the intersect of X-axis and Y-axis was set at the limit of detection (LOD). More details are provided in S5 Fig. (D) Viral sgRNA copies in lung lobes, expressed per ng RNA. For all animals, specimens of 6 different lung lobes (all lobes except accessory lung lobes) were tested for sgRNA levels, expressed relative to cellular RNA. Each symbol represents one lung lobe. Lines represent median values; dotted line represents the limit of detection. While comparison of all 3 groups by Kruskal Wallis test was not significant (p = 0.15), combining the 2 mAb groups for comparison with the control group revealed that mAb treatment was associated with reduced tissue sgRNA levels (p = 0.05, one-sided Mann-Whitney). In addition, mAb-treated animals had a higher proportion of negative lung samples (p = 0.027, Chi-square for comparison of the 3 groups; p = 0.013, two-sided Fisher’s exact test for comparison of control animals versus the combined mAb groups). More detailed data are provided in S6 Fig.

Analysis of sgRNA in nasal and oropharyngeal swabs demonstrated that treatment with CoV-2-mAb at 1 day after infection resulted in inhibition of virus replication in the upper respiratory tract, as reflected in accelerated decline of sgRNA levels (Figs 5A and 5B and S7; ANOVA p = 0.016 and 0.006, for nasal and oropharyngeal sgRNA, respectively). Consistent with variable levels of fluid recovery during bronchoalveolar lavage (BAL), the levels of virus in BAL were more variable, and showed a trend towards a more rapid decline of sgRNA levels in CoV-2-mAb-treated animals, that did not reach statistical significance (Fig 5C).

In contrast to nasal and oropharyngeal swabs we found high variability (often ranging from undetectable to high sgRNA levels) between different lung lobe specimens in all animals (S6 Fig). Despite this high level of variability, comparison of sgRNA levels in lung lobes of the different treatment groups revealed that the CoV-2-mAb-treated animal groups had reduced levels of sgRNA in comparison to the control group (Fig 5D).

### CoV-2 monoclonal antibody treatment reduces lung pathology

Evaluation of infection-induced lung pathology required taking into consideration the multifocal to locally extensive highly random distribution of the lesions and absence of distinct borders. Moreover, in order to accurately evaluate the severity of the lesions, at least x40 magnification was required.

The most striking and consistent lesion in the lungs of SARS-CoV-2 infected macaques was a mild to moderate interstitial pneumonia that frequently radiated out from the terminal bronchioles and was sometimes located in subpleural regions. Histology in these regions showed that alveolar septae were expanded by small to moderate numbers of macrophages and occasional neutrophils. Alveolar spaces variably contained macrophages, a few neutrophils and occasional cellular debris. In lungs with moderate to more severe changes there were occasional hyaline membranes and type 2 pneumocyte hyperplasia (indicative of more severe type 1 pneumocyte injury) and rare interstitial fibrosis (indicative of a slightly more chronic and resolving lesion). Similar lesions have been reported by others in macaques [30–33].

**Fig 6:**
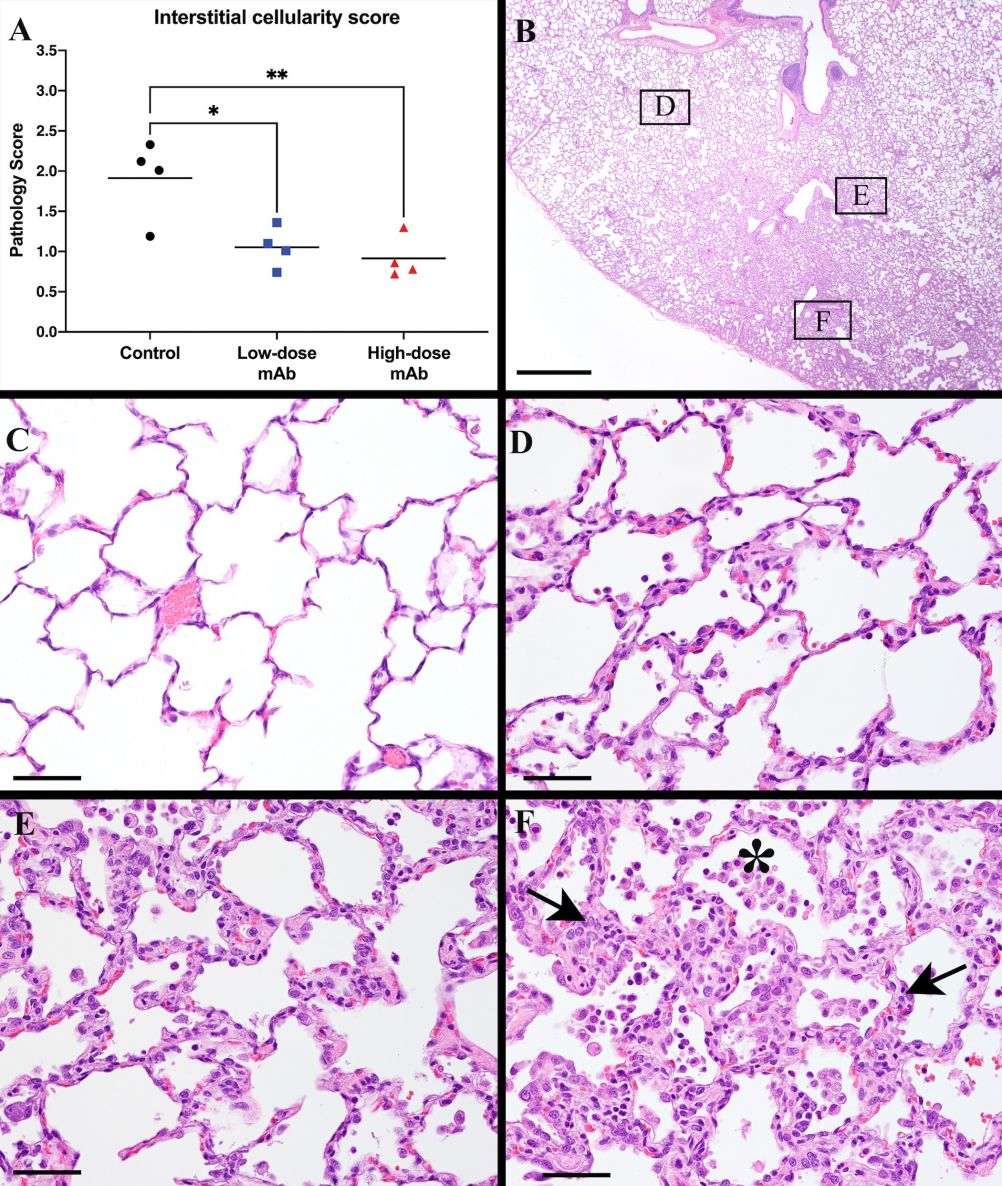
Reduced pulmonary lesions in mAb-treated animals. (A) Interstitial cellularity was evaluated on 3 lung lobes and an average score, weighted for fields (approximately 450–675 fields per animal) was tabulated as outlined in the methods section. Lines indicated mean values. The 2 animal groups that received anti-SARS-CoV-2 mAbs had significantly lower scores than the control group (ANOVA p = 0.007; Dunnet’s multiple comparison relative to control group: * *p* = 0.014; ** *p* = 0.006). For other histology markers that were evaluated, see S9 Fig. (B) SARS-CoV-02 infection induces multifocal to diffuse poorly demarcated lesions of variable severity that are difficult to identify at low power. Bar = 1 mm. (C) Normal alveolar regions. Note thin delicate alveolar septae and no to rare intra-alveolar macrophages Bar = 50 μm**. (**D), (E) and (F) are high power views of the alveolar regions from 3 sites marked in (B) illustrating the range of alveolar lesions identified from mild (D) with little interstitial expansion by inflammatory cells to moderate (E) and severe (F) alveolar interstitial expansion by a cellular infiltrate (arrow) accompanied by abundant alveolar macrophages and some neutrophils (asterisk). Bar = 50 μm.

As outlined in the methods and S8 Fig, a scoring system was developed to tabulate an interstitial cellularity score. Accordingly, the CoV-2-mAb-treated groups had significantly reduced interstitial pneumonia scores in comparison to the control group (Fig 6). The CoV-2-mAb-treated groups also fared better than the control group when examining other markers of lung pathology outlined in the methods (S9 Fig).

Small polypoid or nodular proliferative lesions were occasionally seen in the alveoli of the control animals. These consisted of an organizing core of fibrin with early fibroblast infiltration, covered with attenuated epithelial cells attempting re-epithelialization. However, none of these polypoid alveolar lesions were seen in the CoV-2-mAb-treated groups. Other lesions described below were seen in some animals but did not necessarily correlate with the severity of the interstitial pneumonia. These lesions included bronchiolitis with some epithelial necrosis, loss or attenuation, and peribronchial mixed inflammation which included macrophages, neutrophils and eosinophils, and occasional epithelial syncytial cells (previously reported to be pancytokeratin positive and CD68 negative, confirming their epithelial cell origin [34]). Similar inflammatory infiltrates were sometimes also detected in the submucosa of larger airways. In some animals there was marked perivascular cuffing, particularly of medium and small sized vessels (predominantly lymphocytes and neutrophils) which did not necessarily correlate with the severity of the interstitial pneumonia. There was minimal to mild BALT hyperplasia. One control animal (Co-3, the same animal that had the most severe clinical score) had a severe pleuritis.

Altogether, the histological data further demonstrate the therapeutic efficacy of the combination of CoV-2-mAbs.

### Multivariable analysis of correlates of efficacy

**Fig 7:**
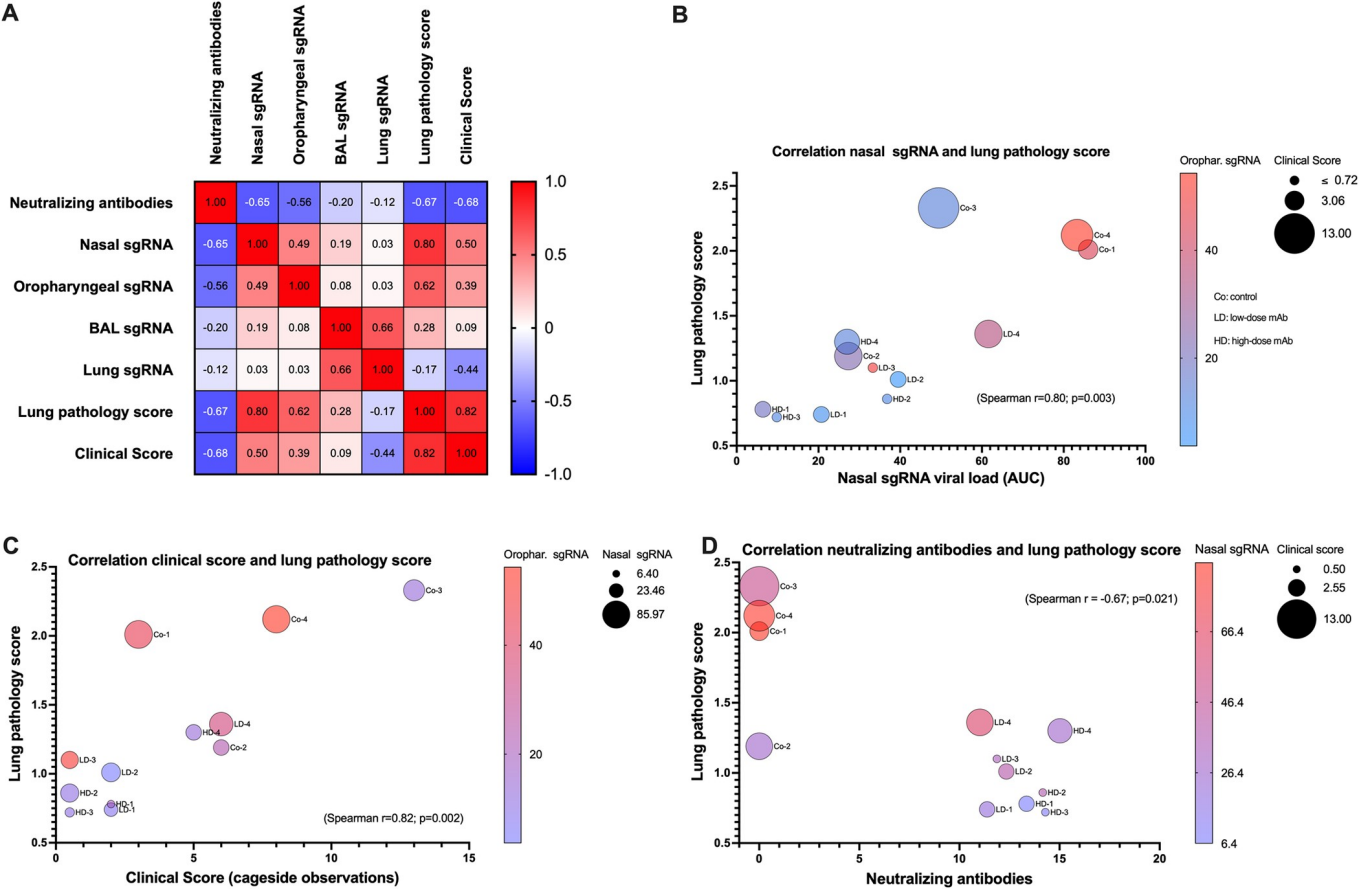
Multivariable correlation analysis. (A). Spearman r correlation matrix in heatmap format. For this analysis, neutralizing antibody levels represent the area-under-the-curve (AUC) of log-transformed NT_90_ values (Fig 2); nasal, oropharyngeal and BAL sgRNA values are based on AUC of the data in Fig 5A–5C. Lung sgRNA levels for each animal represent the geometric mean sgRNA of the 6 lung lobes that were tested (using data of S6 Fig). Lung pathology scores and clinical scores are the data presented in Figs 3D and 6A). Bubble plots (B) through (D) demonstrate 3 markers with highest Spearman r correlation values on the X- and Y-axis, with dot size and color representing other parameters that had moderate to high correlation with these 2 markers. The labels next to each dot indicate the individual animals (4 animals per group).

A multivariable analysis was performed on the main markers that individually reflected the therapeutic efficacy of the mAbs (Fig 7). With the caveat of the small group sizes, significant correlations were observed between lung pathology scores and clinical scores (Spearman r = 0.82; p = 0.002), lung pathology scores and neutralizing antibody titers (Spearman r = -0.67, p = 0.021), and lung pathology scores and virus replication in upper respiratory tract (Spearman r = 0.80; p = 0.003, and r = 0.62, p = 0.035 for nasal and oropharyngeal sgRNA levels, respectively) (Fig 7). In contrast, sgRNA viral loads in BAL and lung tissues correlated poorly with lung pathology and clinical scores, which further indicate the variability and irregular distribution of virus replication in the lower respiratory tract (Fig 7).

## Discussion

The current study provides further support to the use of potent neutralizing mAbs to treat early SARS-CoV-2 infection. We find that administration of a combination of CoV-2 mAbs, C135-LS and C144-LS to rhesus macaques, one day after SARS-CoV-2 inoculation, improved clinical scores, reduced virus replication in upper and lower respiratory tract, and reduced lung pathology in comparison to the placebo-treated controls. That most endpoint differences reached statistical significance is remarkable, considering the small group sizes, the very high virus inoculum dose, and the generally mild-to-moderate disease course in this animal model, but testifies to the potency of these antibodies. The lack of differences between the two CoV-2-mAbs treatment arms, 40 vs 12 mg/kg total dose, is consistent with studies in infected Syrian hamsters where doses of 4 mg/kg delivered intraperitoneally were highly effective for therapy [35]. Altogether, these data support ongoing and future clinical trials with C135-LS/C144-LS and other mAbs to treat SARS-CoV-2 infected patients during the early stages of infection to reduce virus replication, halt disease progression and reduce mortality. Our data do not apply to treatment during later stages of disease, where success is likely to be contingent on treatments that directly reduce inflammation.

A potential drawback of the experimental design of the current and other post-infection therapy studies in animal models is that antiviral interventions (such as antibodies, convalescent plasma, or antiviral drugs) are generally given within 12–24 hours after virus inoculation [15,16,23], which does not mirror SARS-CoV-2 exposed people, where diagnosis of infection and initiation of any treatment is likely to be several days or more after infection. However, consideration of the difference in viral replication kinetics between naturally infected people and experimentally infected macaques provides support to the relevance of data derived from this experimental design. Natural infection of people is likely to be initiated with a low amount of virus in the upper respiratory tract and larger airways; while virus replication can remain limited to those areas, in a proportion of people it will disseminate to the lower respiratory tract and cause interstitial pneumonia [36]. In contrast, experimental inoculation of macaques with SARS-CoV-2 has an accelerated time course, beginning with a large inoculum administered intranasally and intratracheally to directly seed infection of the upper and lower respiratory tract. Consistent with previous observations by us and other groups [15,21,30,37], we find that virus replication in the respiratory tract of infected macaques peaked within the first ~2 days. Thus, our experimental design mimics treatment of SARS-CoV-2 infected people initiated around the time of peak virus replication.

Animals treated with a combination of two CoV-2-mAbs had significantly improved outcomes, including reduced virus replication in both the upper and lower respiratory tract. In contrast, remdesivir treatment of SARS-CoV-2 infected macaques, initiated 12 hours after virus inoculation, reduced virus replication only in the lower respiratory tract but had no detectable effect on virus replication in the upper respiratory tract [23]. Although symptoms associated with virus replication in the upper respiratory tract are generally absent or mild (e.g., sore throat, runny or congested nose), the observation that mAb therapy rapidly reduced this virus replication is clinically relevant because (i) it is expected to reduce transmission to other people, and (ii) it may reduce complications in the central nervous system, as evidence indicates that SARS-CoV-2 virus can invade the brain via the olfactory route [38,39]. The effects of antibody-based therapies on these clinical implications warrant further research.

COVID-19 morbidity and mortality are associated with respiratory failure due to inflammation. Our findings highlight some of the challenges of assessing the therapeutic effects of an intervention on virus replication and pathology in the lung. The distribution of viral RNA and lung histopathology revealed that sites of virus replication are random and unpredictable. The reasons for this are likely multi-factorial, but may include the method of virus inoculation, with virus suspended in fluid (instead of being aerosolized), which determines the initial seeding and subsequent distribution over the lung lobes. Moreover, viral antigen can be found in both lesional and non-lesional tissues [34], suggesting that viral antigen alone without concurrent histopathology is not a reliable surrogate endpoint to indicate the relative success of an intervention.

Challenges encountered when evaluating histology of SARS-CoV-2 infected lung include (i) the extremely random distribution pattern of the lung lesions, (ii) lack of distinct borders between affected and normal lung parenchyma regions, and (iii) the observation that the severity of lesions was difficult to evaluate at low-power microscopy. These difficulties necessitated the development of the scoring system described in this report. While being quite labor-intensive, this scoring system systematically and in an unbiased way picks out the high magnification fields to be scored. This prescriptive selection process removes the tendency to only focus on the most severe or least severe lesions in a section which would otherwise bias the scoring. Furthermore, as different features of the disease are more prominent at different time points, the scoring system needed to be relevant to the stage of disease in this model. Similar to findings in hamsters [40], interstitial pneumonia was the most prominent feature that was consistent throughout all SARS-CoV-2 infected macaques at day 7; hence our focus on scoring this lesion.

Clinical signs of dyspnea in humans can be attributed to interstitial pneumonia and alveolar compromise which is mainly due to injury to type 1 and type 2 pneumocytes. The relationship between alveolar damage and dyspnea is supported by computed tomography (CT), which identified early lesions indicative of an alveolar pattern of disease in a large study from China in which 19% of patients presented with shortness of breath [41]. Interestingly, chest imaging has also revealed abnormalities in people who are PCR+ for SARS-CoV-2 but asymptomatic [42]. The most prominent lesion in severe human disease is diffuse alveolar damage with extensive epithelial desquamation, hyaline membrane formation and alveolar edema. Not surprisingly, reports of histological findings in humans with early infection or disease that did not become severe are sparse. In the small number of reports where lesions were identified incidentally, lesions were variable and of unknown duration, although most appear to be more chronic [43–45]. The lesions in this rhesus macaque model may provide clues as to the appearance of milder and often asymptomatic human disease and indicate considerable interstitial inflammation present in alveolar septae despite lack of progression to extensive (diffuse) alveolar damage with hyaline membrane formation. This lack of damage is probably due to more limited injury to type 1 pneumocytes.

An appropriate lung histopathology scoring system in animal models of COVID-19 is exceptionally important when evaluating therapeutic interventions, particularly when interventions are less potent and/or are administered later in the disease course. If not properly evaluated, moderate efficacy may be missed especially given the inherent small group sizes of nonhuman primate studies. The elaborate scoring system may also address an often common criticism of the small group sizes of nonhuman primate studies: scoring a few hundred microscopic lung fields in a limited number of macaques, in which lung lesions are randomly dispersed, may provide information that is scientifically equivalent to evaluating a few lung sections in a large number of animals.

In conclusion, administration of a combination of C135-LS and C144-LS, one day after SARS-CoV-2 infection, had significant therapeutic efficacy as defined by improved clinical outcome, reduced virus replication in upper and lower respiratory tract, and reduced lung inflammation. These results support the further clinical development of these and other potent mAbs. In addition, the study highlights some of the challenges of the animal model in evaluating the effects of interventions on the lungs. Some of these issues can be resolved by the pathology techniques employed, further strengthening the value of this animal model as a resource to test therapeutic strategies against SARS-CoV-2 or other respiratory pathogens that may cause future pandemics.

## Materials and methods

### Ethics statement

The study was approved by the Institutional Animal Care and Use Committee of the University of California, Davis (study protocol 21735).

### Animals and care

All 12 rhesus macaques (Macaca mulatta) in the study were young adults (3.4 to 6 years of age), born and raised in the breeding colony of the California National Primate Research Center (CNPRC), which is negative for type D retrovirus, SIV and simian lymphocyte tropic virus type 1. Prior to enrollment, animals were confirmed to be seronegative and RT-PCR negative for SARS-CoV-2 and were kept in a special barrier room prior to study initiation. Animals were moved into the animal biosafety level 3 (ABSL-3) facility just before virus inoculation. Each of the 3 study arms (4 animals) had equal sex distribution (2 males and 2 females).

The CNPRC is accredited by the Association for Assessment and Accreditation of Laboratory Animal Care International (AAALAC). Animal care was performed in compliance with the 2011 *Guide for the Care and Use of Laboratory Animals* provided by the Institute for Laboratory Animal Research. Macaques were housed indoor in stainless steel cages (Lab Product, Inc.) whose sizing was scaled to the size of each animal, as per national standards, and were exposed to a 12-hour light/dark cycle, 64–84°F, and 30–70% room humidity. Animals had free access to water and received commercial chow (high protein diet, Ralston Purina Co.) and fresh produce supplements.

### Virus and inoculations

A virus stock of a Washington isolate was obtained from BEI Resources (SARS-CoV-2 2019-nCoV/USA-WA1/2020; NR-52352; Lot/Batch # 70033952). The titer of this stock was 10^6^ PFU/ml. Animals were inoculated with a total of 2.5 ml (2.5x10^6^ PFU), of which 2 ml was administered intratracheally via a 8 fr PVC feeding tube, and 0.5 ml was administered intranasally (0.25 ml per nostril).

### Monoclonal antibody administration

All monoclonal antibodies were administered via slow intravenous infusion approximately 24 hours after virus inoculation. The SARS-CoV-2 neutralizing monoclonal antibodies C135-LS and C144-LS were administered in combination, either at doses of 6 or 20 mg/kg for each antibody (low-dose and high-dose mAb group). A control group received a non-SARS-CoV-2 monoclonal antibody (HIV neutralizing monoclonal antibody 10-1074-LS) at a dose of 20 mg/kg. All monoclonal antibodies had the LS mutation in the Fc region to extend their half-lives.

### Clinical observations and sample collections

Daily cage-side clinical monitoring was performed by a veterinarian who was blinded to the group assignments, and included recording of responsiveness, discharge, respiratory rate and character, evidence of coughing/sneezing, appetite, stool quality. A score was tabulated for each of these parameters, and a total score was calculated for each animal per day (S3 Table). When animals had to be sedated for procedures, additional clinical assessments (including rectal temperature, respiration, spO2, heart rate, skin turgor/hydration) were recorded by the same veterinarian (S4 Table). Animals were sedated with ketamine HCl (10 mg/kg IM) for the clinical assessment. Dexmedetomidine (15 μg/kg IM) was administered after clinical assessments to facilitate sampling, and midazolam (0.25–0.5 mg/kg IM) was added as needed. Oxygen saturation was obtained by pulse oximetry with a Radical 7 (Masimo, Irvine, CA). Blood pressure was obtained via oscillometry with a Vet25 and an appropriately sized cuff according to the manufacturer’s instructions (SunTech, Morrisville, NC).

Blood was collected via peripheral venipuncture. Complete blood counts were performed on EDTA-anticoagulated blood samples, with electronic cell counts performed on a Pentra 60C+ analyzer (ABX Diagnostics) or Vet abc™ (SCIL Animal Care); differential cell counts were determined manually. EDTA anti-coagulated blood was also used for immunophenotyping and, after centrifugation, the collection of plasma. Blood tubes without coagulant and CPT™ vacutainer tubes were also collected for processing via centrifugation (900xg for 10 minutes) for serum and peripheral blood mononuclear cells, respectively. Plasma and serum aliquots were stored at -70°C until further processing.

Nasopharyngeal and oropharyngeal secretions were collected with FLOQSwabs™ (Copan), placed in a vial with DNA/RNA Shield™ solution (Zymo Research), and stored at -70°C until further processing.

Bronchoalveolar lavages (BAL) were performed using a 20F rubber feeding tube with instillation of 20 ml sterile physiologic saline followed by aspiration with a syringe. BAL samples were spun in the lab, and cell pellet and aliquots of supernatant were cryopreserved at -70° C.

At the end of the study, animals were euthanized, and a full necropsy was performed for tissue collection. Tissues were collected both for viral load analysis in RNA*later* (Invitrogen), and in 10% neutral-buffered formalin for histology. Selected lung lobes were dissected to obtain both peripheral and central samples for viral load analysis; other lung lobes, after removal of a clamped peripheral section for viral load analysis, were drip-infused with 10% neutral-buffered formalin (see further).

### Collection and evaluation of radiographs

Radiographs were obtained with a HF100+ Ultralight imaging unit (MinXRay, Northbrook, IL) at 50 kVp, 40mA, and 0.1 sec. Ventrodorsal, dorsoventral, R lateral, and L lateral radiographs were obtained prior to inoculation and every other day after virus inoculation (days 1, 3, 5, and 7). Radiographs were scored for the presence of pulmonary infiltrates by a board-certified veterinary radiologist, who was blinded to the experimental group and time point, according to a standard scoring system (0: normal; 1: mild interstitial pulmonary infiltrates; 2: moderate pulmonary infiltrates perhaps with partial cardiac border effacement and small areas of pulmonary consolidation; 3: severe interstitial infiltrates, large areas of pulmonary consolidation, alveolar patterns and air bronchograms). Individual lobes were scored and scores per animal per day were totaled.

### Viral load determination by RT-qPCR analysis

Quantitative real-time PCR assays were developed for detection of full-length genomic vRNA (gRNA), sub-genomic vRNA (sgRNA), and total vRNA. RNA was extracted from swabs preserved in DNA/RNA Shield using the Quick-RNA Viral Kit (Zymo Research). BAL cell pellets were processed directly in TRIzol-LS reagent (ThermoFisher) and total RNA purified using the Qiagen RNeasy Mini Kit (Qiagen). Tissues preserved in RNAlater were transferred to Qiazol, and homogenized with a 7mm stainless steel bead in a TissueLyser (Qiagen), and processed using the Qiagen RNeasy Mini Kit. Following DNase treatment with ezDNase (ThermoFisher), complementary DNA was generated using random hexamers, Superscript IV Reverse Transcriptase (ThermoFisher) in the presence of RNaseOUT (ThermoFisher). A portion of this reaction was mixed with QuantiTect Probe PCR Kit and optimized concentrations of gene specific primers. All reactions were run on a Quantstudio 12K Flex real-time cycler (Applied Biosystems). gRNA was quantified by targeting orf1a-nsp4 using primers orf1a_F7 (GTGCTCATGGATGGCTCTATTA) and orf1a_R7 (CGTGCCTACAGTACTCAGAATC), with probe orf1a_P7 (/56-FAM/ACCTACCTT/ZEN/GAAGGTTCTGTTAGAGTGGT/3IABkFQ/). sgRNA was quantified using primers sgLeadSARSCoV2_F (CGATCTCTTGTAGATCTGTTCTC) and wtN_R4 (GGTGAACCAAGACGCAGTAT), with probe wtN_P4 (/56-FAM/TAACCAGAA/ZEN/TGGAGAACGCAGTGGG/3IABkFQ/). Total vRNA was quantified using primers wtN_F4 (GTTTGGTGGACCCTCAGATT) and wtN_R4, with probe wtN_P4. Standard curves generated from PCR amplicons of the qPCR targets were used to establish line equations to determine RNA copies/mL or copies/ug RNA. A *macaca* housekeeping gene PPIA was used as a reference (Taqman Gene Expression Assays Rh02832197_gH, PPIA; Applied Biosystems PN4351370). The amount of viral RNA relative to PPIA mRNA was expressed by tabulating the difference in Ct values for each sample.

### Determination of neutralizing antibody titers

The pseudotype neutralization assays were performed as previously described [11]. In brief, macaque plasma was four-fold serially diluted and then incubated with SARS-CoV-2 pseudotyped HIV-1 reporter virus for 1 h at 37°C. The plasma/pseudotyped virus mixture was then added to HT1080/ACE2.cl14 cells. After 48 h, cells were washed with PBS, lysed with Luciferase Cell Culture Lysis reagent (Promega) and Nanoluc Luciferase activity in lysates was measured using the Nano-Glo Luciferase Assay System (Promega) and a Glomax Navigator luminometer (Promega). The relative luminescence units were normalized to those derived from cells infected with SARS-CoV-2 pseudotyped virus in the absence of plasma. The half-maximal inhibitory concentrations (NT_50_) were determined using four-parameter nonlinear regression (least squares regression method without weighting) (GraphPad Prism).

### Measurement of cytokines and chemokines in plasma

A Procarta Plex immunoassay (ThermoFisher Scientific) was used to measure interferon(IFN)-alpha, IFN-gamma, IL-1beta, IL-6, IP-10, I-TAC, MCP-1. Interferon-beta was measured using a monkey interferon-beta1 ELISA kit (Mybiosource) as per manufacturer’s instructions.

### Serum biochemistry

Biochemistry analysis on serum samples was performed using Piccolo® BioChemistry Plus disks, that were run on the Piccolo® Xpress Chemistry Analyzer (Abbott), according to the manufacturer’s instructions. This panel includes alanine aminotransferase (ALT), albumin, alkaline phosphatase (ALP), amylase, aspartate aminotransferase (AST), C-reactive protein, calcium, creatinine, gamma glutamyltransferase (GGT), glucose, total protein, blood urea nitrogen (BUN), and uric acid.

### Measurement of T cell responses by flow cytometry

PBMCs (1x10^6^ cells) from each study animal were stained with a panel of fluorophore conjugated monoclonal antibodies against the following surface antigens: CD3, CXCR3, CD20, PD1, CCR6, CXCR5, CCR4, CD4, CCR5, CD69, CD95, CD8, and dump channel (CD20, and live/dead) markers for 30 minutes at 4°C. Cells were subsequently lysed with 1000μL of FACS lyse (BD Biosciences, USA), washed twice with 1X FACS buffer. Cells were fixed and permeabilized with 100μL of FoxP3 fixation (BD Biosciences, USA) for 10 minutes at dark at room temperature, followed by washing with 1X FoxP3 wash buffer. Intracellular staining was performed with Ki-67 and Granzyme B for 45°C minutes at room temperature. Stained cells were washed and re-suspended in FACS buffer. Fluorescence was measured on the same day using a BD FACSymphony with FACS Diva version 8.0.1 software. Compensation, gating, and analysis were performed using FlowJo (Versions 9 and 10). Antibody/reagent details are given in S5 Table.

### Euthanasia and evaluation of pathology

All animals were euthanized at day 7 after infection with an overdose of pentobarbital and subjected to a full necropsy under BSL-3 conditions. The lungs were harvested and each lobe separated. The caudal lobes, right cranial lobe and middle portion of the left cranial lobe were cannulated with 18-gauge blunt needles. A small peripheral section of the cannulated lobe was clamped off and removed to be saved for measuring viral RNA levels. Then these 3 lobes were slowly infused with neutral buffered formalin at 30 cm fluid pressure. Once fully inflated (approx. 30 mins) the main bronchus was tied off and the lungs were placed in individual jars of formalin and fixed for 72 hours. Then they were sliced from the hilus towards the periphery into slabs approximately 5mm thick. Each slab was placed into a cassette recording its position in the stack and with further division of the slab into smaller pieces if required to fit into the cassette. Tissues were then held in 70% ethanol until processing. A full set of remaining tissues was harvested at necropsy, trimmed into cassettes and then fixed in 10% neutral buffered formalin for 72 hours before transfer into 70% ethanol. The lung and all other tissues were sent for routine tissue processing and paraffin embedding followed by sectioning at 5 μm and generation of H&E stained slides.

Slides from every other slab of the right cranial, middle portion of the left cranial and the most severely affected caudal lobe were examined independently by 2 ACVP board certified pathologists; 25 randomly selected x40 fields from each slide were evaluated for the severity of the interstitial inflammation (mostly mononuclear cells sometimes with neutrophils) and this was graded on a scale of 0–4, as described in S8 Fig. Altogether, this resulted in evaluating between 450 to 675 microscopic fields (at x40 magnification) from 3 lung lobes per animal. Then a weighted-average overall score was calculated that takes into account the number of readings per lung lobe (i.e., larger lung lobes, having more readings, contribute more to the overall score than smaller lobes).

The numbers of alveolar macrophages, alveolar neutrophils, interstitial fibrosis and type 2 hyperplasia were assessed and scored for each slide, as described in S6 Table. The other tissues were also examined by an ACVP Diplomate but no other significant lesions were identified.

### Statistical analyses

Statistical analyses were performed using Prism version 9 (GraphPad), with selection of the test as outlined in the results. P values of <0.05 were considered statistically significant.

## Supporting information

S1 Fig Clinical measurements collected at time of sedation. Red and black arrows indicate time of virus inoculation and monoclonal antibody administration on days 0 and 1, respectively. (A) Body weight remained stable. (B) Rectal temperature; horizontal line indicates the cut-off of 103° F, above which ketoprofen treatment was administered. (C) Respiratory rate; the horizontal line indicates a cut-off value of 55 (per minute) as upper normal range. (D) Oxygen saturation measured by pulse oximetry; the horizontal line indicates 95% as the lower end cut-off of the normal range. (E) Total clinical scores, including the markers not graphed above but included in S4 Table. (F) Total sedated scores per group from day 0–7 (lines represent median values) did not show significant differences.
(TIF)

S2 Fig Time course of cytokines and chemokines in plasma of SARS-CoV-2 inoculated animals. Cytokines and chemokines were measured in plasma using established Luminex-based or ELISA methodology (see methods section). The graphs show all markers with at least one value above the limit of detection. Other cytokines and chemokines that were measured (IL-1beta, IL-6, IP-10) were below the limit of detection throughout the 7-day time course of the experiments. Red and black arrows indicate time of virus inoculation and monoclonal antibody administration on days 0 and 1, respectively.
(TIF)

S3 Fig Time course of serum chemistry markers in SARS-CoV-2 inoculated animals. Biochemistry analysis on serum samples was performed using Piccolo® BioChemistry Plus disks. (A) through (C) present C-reactive protein (CRP), alanine aminotransferase (ALT), and aspartate aminotransferase (AST), which showed transient changes during the early stages of infection regardless of the study group. Other markers in the panel did not show any obvious changes. Red and black arrows indicate time of virus inoculation and monoclonal antibody administration on days 0 and 1, respectively.
(TIF)

S4 Fig Increase in proliferating T cells in circulation following SARS-CoV-2 infection. (**A**) Gating strategy to identify CD4 and CD8 T cells. (**B**) shows Ki-67^+^ CD4 T cells and expression of PD-1, CCR6, CXCR3 on proliferating CD4 T cells and respective kinetics. (**C**) shows induction of Ki-67^+^ CD8 T cells, expression of PD-1, and association between proliferating CD4 T and CD8 T cells. LD and HD indicate low-dose and high-dose mAb, respectively.
(TIF)

S5 Fig Viral RNA levels in nasal swabs, oropharyngeal swabs and BAL cell pellets. Nasal swabs (A), oropharyngeal swabs (B) and BAL cell pellets (C) were tested by RT-qPCR for total, genomic and subgenomic viral RNA, and the housekeeping gene PPIA mRNA. Viral RNA levels are expressed relative to PPIA mRNA by graphing the difference in Ct values. For each sample type, the top figures show the individual data (with the intersection of X-axis and Y-axis set near the limit of detection); the bottom figures display the median values per group. Red and black arrows indicate time of virus inoculation and monoclonal antibody administration on days 0 and 1, respectively.
(TIF)

S6 Fig Viral RNA levels in lung tissues. Specimens of 6 lung lobes were tested by RT-qPCR for total, genomic and subgenomic viral RNA (expressed relative to cellular RNA). For 3 lung lobes, only a peripheral section (removed before infusion of the rest of the lobe with formalin; see materials and methods) was available for viral load testing; for the other 3 lung lobes, a central specimen was tested for viral load. The pattern of detectable viral RNA reflected random dispersion among the lung lobes, with no discernible predilection of virus replication for left versus right lung lobes, or peripheral versus central lung tissue. The vertical line separates right from left side lung lobes.
(TIF)

S7 Fig Effect of monoclonal antibody treatment on sgRNA kinetics in nasal and oropharyngeal swabs of SARS-CoV-2 infected macaques. A weighted average analysis was performed on the sgRNA data in nasal and oropharyngeal swabs (S5 Fig) to calculate the relative decline of viral RNA (relative to cellular mRNA in the sample) from day 1 to day 7. For each animal, the AUC of relative sgRNA per cellular mRNA over time (S5 Fig) was tabulated using day 1 as baseline, and then divided by 6 days to get the weighted average in the decline of sgRNA over the 6-day time period. Lines indicate mean values. Statistical analysis revealed significant effects of monoclonal antibody treatment. For panel A, ANOVA: p = 0.016; Dunnett’s multiple comparison test: Control versus low-dose mAb: adjusted p = 0.018; Control versus high-dose mAb: adjusted p = 0.021. For panel B, ANOVA: p = 0.006; Dunnett’s multiple comparison test: control versus low-dose mAb: adjusted p = 0.02; control versus high-dose mAb: adjusted p = 0.004.
(TIF)

S8 Fig Interstitial cellularity scoring. Interstitial cellularity score assigned to 25 unbiased random x40 fields per slide is based on the number of cells expanding the alveolar interstitium. Grade 1 (A) 1–2 cells thick, grade 2 (B) 3–4 cells thick, grade 3 (C) 5–6 cells thick, grade 4 (D) >6 cells thick. Bar = 50 μm. The score is allocated according to the most severe region within the field. Total score per animal is based on a weighted average of approximately 450 to 675 fields scored per animal.
(TIF)

S9 Fig Additional lung histology scores. In addition to evaluation of lung septal cellularity as primary marker of interstitial pneumonia (see **Fig 6**), lung sections were also scored for other evidence of inflammation, injury and repair, including presence of alveolar macrophages (A), alveolar neutrophils (B), septal fibrosis (C), type 2 hyperplasia (D) and pleuritis (E) (as described in S6 Table). For each graph, p values represent Kruskal-Wallis test for comparison of the 3 study groups. C, LD and HD represent control, low-dose and high-dose SARS-CoV-2 mAb groups, respectively. For graph E, only one control animal (Co-3) had pleuritis.
(TIF)

S1 Table Neutralization titers and antibody concentrations in serum of macaques. Animals were inoculated with SARS-CoV-2 on day 0, and antibodies were infused on day 1. 50% and 90% neutralization titers (NT50 and NT90) in serum were measured by a RVPN assay. Concentrations of CoV-2 mAbs were calculated based on the NT_50_ and NT_90_ values of the RVPN curves for each sample and using the neutralization activity of the combination of antibodies. The mean values for the 2 CoV-2 mAb groups from day 2 to day 7 were calculated (bottom section) and reflect the ~3-fold differences in dosage between the 2 treatment groups.
(DOCX)

S2 Table Summary of radiological scoring. All thorax radiographs were scored blinded by a veterinary radiologist, with scores of 0 to 3 assigned to each of the 7 lung lobes. For each time point, the total score of all lung lobes was tabulated.
(DOCX)

S3 Table Clinical Signs Scoring Criteria for SARS-CoV-2 infected macaques. The above table was adapted from WNPRC COVID scoring sheet (https://openresearch.labkey.com/wiki/Coven/page.view?name=clinical-scoring)), which itself was modified from a previous NHP influenza A virus study to include clinical signs relevant to COVID-19 and respiratory rates for cynomolgus macaques [1–3]. Cageside assessments were performed every day. The highest sum of scores for an animal determined the severity of disease. Clinical disease severity was classified as no clinical illness (0–4), mild (5–9), moderate (10–15), severe (>16). BPM = breaths per minute. 1. Chertow DS, Kindrachuk J, Sheng ZM, Pujanauski LM, Cooper K, Nogee D, Claire MS, Solomon J, Perry D, Sayre P, Janosko KB, Lackemeyer MG, Bohannon JK, Kash JC, Jahrling PB, Taubenberger JK. 2016. Influenza A and methicillin-resistant Staphylococcus aureus co-infection in rhesus macaques—A model of severe pneumonia. Antiviral Res. 129:120–129. doi:10.1016/j.antiviral.2016.02.013. 2. Bolton ID. 2015. Chapter 5—Basic Physiology of Macaca fascicularis. In The Nonhuman Primate in Nonclinical Drug Development and Safety Assessment. J Bluemel, S Korte, E Schenck, GF Weinbauer, editors. Academic Press, San Diego. 67–86. 3. Huang C, Wang Y, Li X, Ren L, Zhao J, Hu Y, Zhang L, Fan G, Xu J, Gu X, Cheng Z, Yu T, Xia J, Wei Y, Wu W, Xie X, Yin W, Li H, Liu M, Xiao Y, Gao H, Guo L, Xie J, Wang G, Jiang R, Gao Z, Jin Q, Wang J, Cao B. 2020. Clinical features of patients infected with 2019 novel coronavirus in Wuhan, China. Lancet. 395:497–506. doi:10.1016/S0140-6736(20)30183-5.
(DOCX)

S4 Table Physical examination under anesthesia scoring criteria. The above table was adapted **from WNPRC COVID scoring sheet (https://openresearch.labkey.com/wiki/Coven/page.view?name=clinical-scoring),** which itself was modified from a previous NHP influenza A virus study to include clinical signs relevant to COVID-19 and respiratory rates for cynomolgus macaques [1–3]. Physical examinations were performed whenever an animal was anesthetized. The highest sum of scores for an animal determined the severity of disease. Clinical disease severity was classified as no clinical illness (0–2), mild (3–7), moderate (8–13), severe (>13). BPM = breaths per minute for respiratory rate and beats per minute for heart rate. 1. Chertow DS, Kindrachuk J, Sheng ZM, Pujanauski LM, Cooper K, Nogee D, Claire MS, Solomon J, Perry D, Sayre P, Janosko KB, Lackemeyer MG, Bohannon JK, Kash JC, Jahrling PB, Taubenberger JK. 2016. Influenza A and methicillin-resistant Staphylococcus aureus co-infection in rhesus macaques—A model of severe pneumonia. Antiviral Res. 129:120–129. doi:10.1016/j.antiviral.2016.02.013. 2. Bolton ID. 2015. Chapter 5—Basic Physiology of Macaca fascicularis. In The Nonhuman Primate in Nonclinical Drug Development and Safety Assessment. J Bluemel, S Korte, E Schenck, GF Weinbauer, editors. Academic Press, San Diego. 67–86. 3. Huang C, Wang Y, Li X, Ren L, Zhao J, Hu Y, Zhang L, Fan G, Xu J, Gu X, Cheng Z, Yu T, Xia J, Wei Y, Wu W, Xie X, Yin W, Li H, Liu M, Xiao Y, Gao H, Guo L, Xie J, Wang G, Jiang R, Gao Z, Jin Q, Wang J, Cao B. 2020. Clinical features of patients infected with 2019 novel coronavirus in Wuhan, China. Lancet. 395:497–506. doi:10.1016/S0140-6736(20)30183-5.
(DOCX)

S5 Table Flow cytometry antibody and reagents. (DOCX)

S6 Table Additional scoring system of lung histology. A single score per slide was given based on the criteria provided above. Total score per animal was provided as a weighted average.
(DOCX)
