# Supplementary figures and images for: Early treatment with a combination of two potent neutralizing antibodies improves clinical outcomes and reduces virus replication and lung inflammation in SARS-CoV-2 infected macaques
Source: PLoS Pathog. 2021 Jul 6;17(7):e1009688. doi: 10.1371/journal.ppat.1009688 (PMC8284825; doi:10.1371/journal.ppat.1009688)

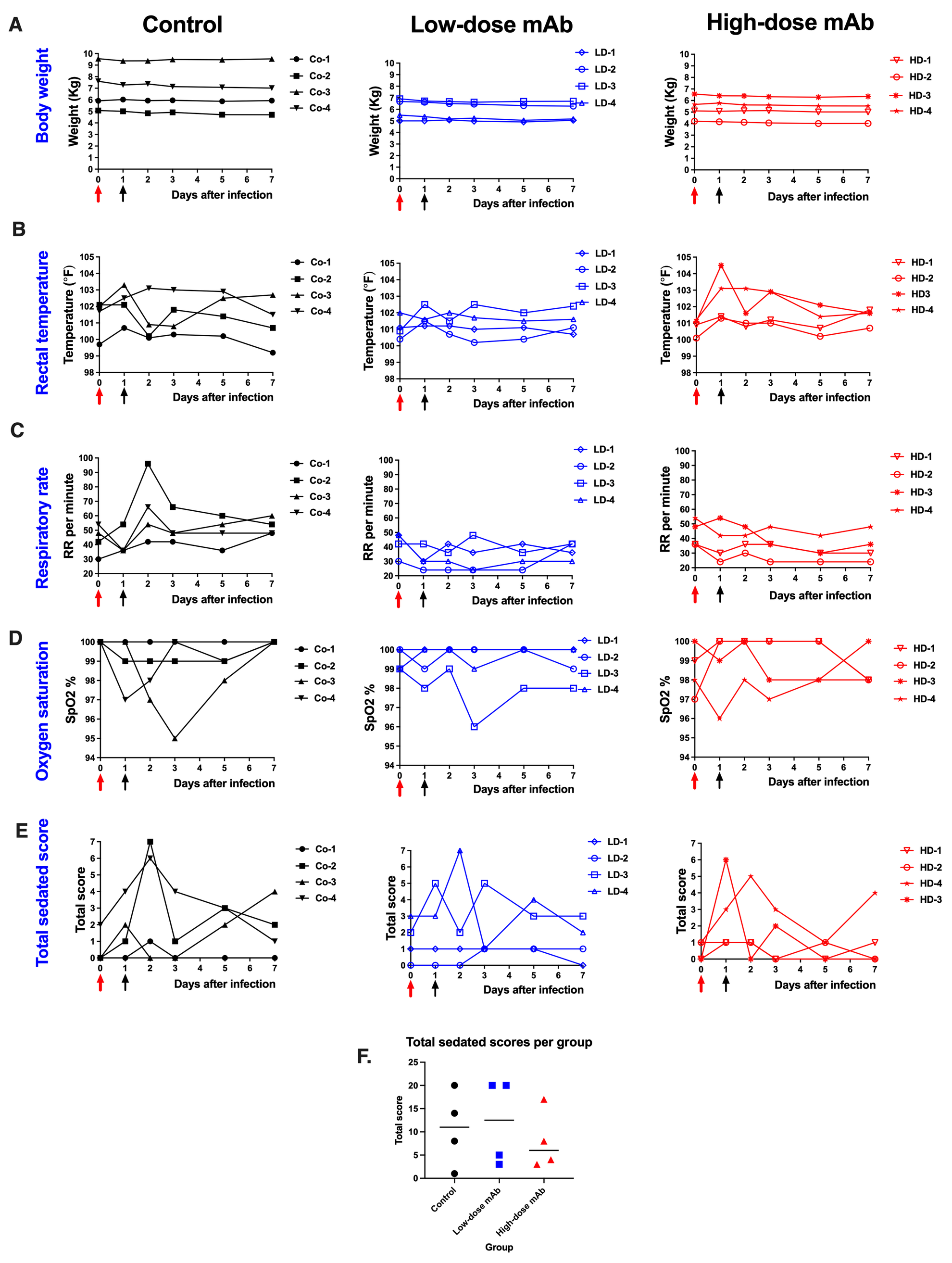

Supplement: S1 Fig — Red and black arrows indicate time of virus inoculation and monoclonal antibody administration on days 0 and 1, respectively. (A) Body weight remained stable. (B) Rectal temperature; horizontal line indicates the cut-off of 103° F, above which ketoprofen treatment was administered. (C) Respiratory rate; the horizontal line indicates a cut-off value of 55 (per minute) as upper normal range. (D) Oxygen saturation measured by pulse oximetry; the horizontal line indicates 95% as the lower end cut-off of the normal range. (E) Total clinical scores, including the markers not graphed above but included in S4 Table. (F) Total sedated scores per group from day 0–7 (lines represent median values) did not show significant differences. (TIF) [file ppat.1009688.s001.tif]

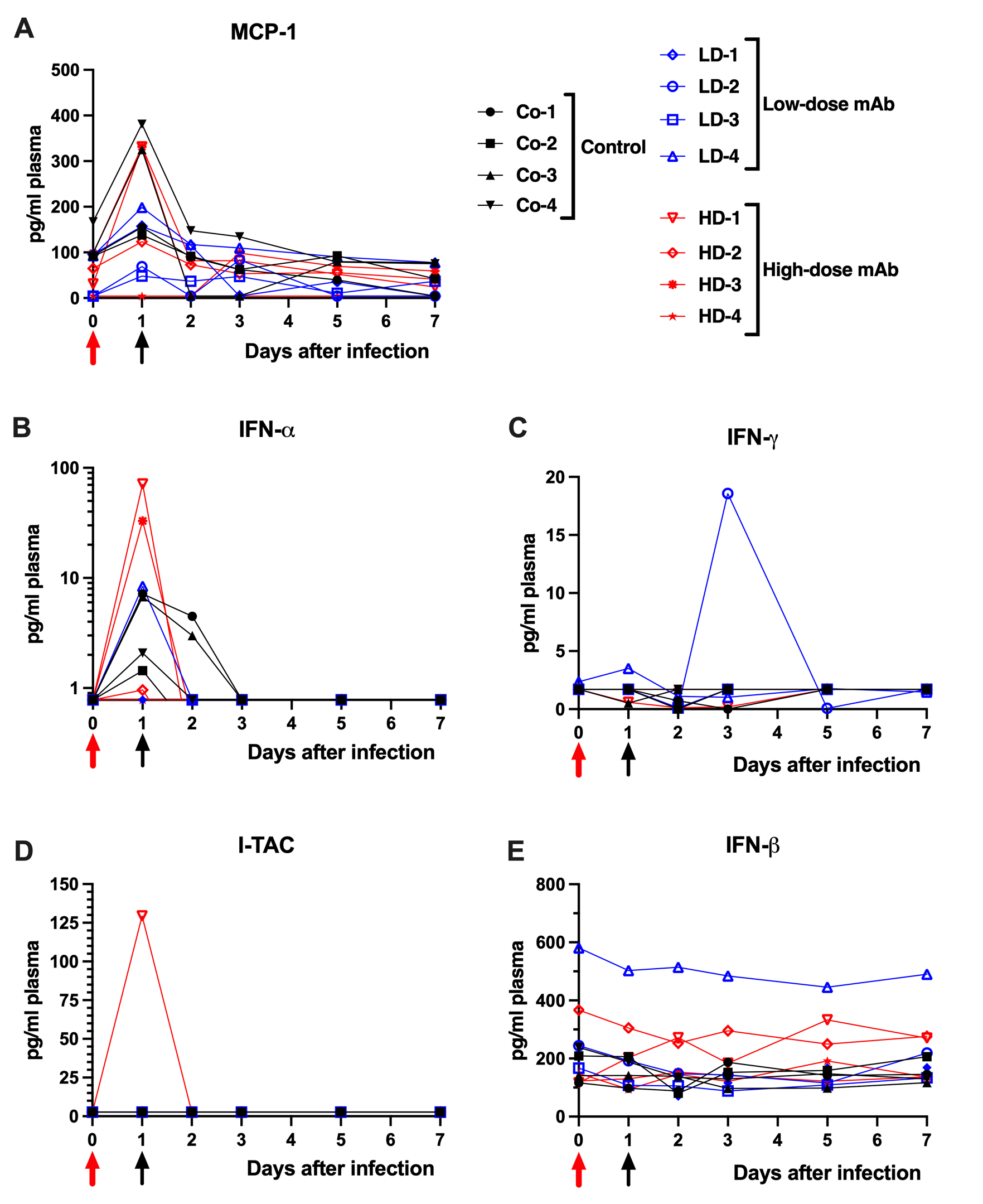

Supplement: S2 Fig — Cytokines and chemokines were measured in plasma using established Luminex-based or ELISA methodology (see methods section). The graphs show all markers with at least one value above the limit of detection. Other cytokines and chemokines that were measured (IL-1beta, IL-6, IP-10) were below the limit of detection throughout the 7-day time course of the experiments. Red and black arrows indicate time of virus inoculation and monoclonal antibody administration on days 0 and 1, respectively. (TIF) [file ppat.1009688.s002.tif]

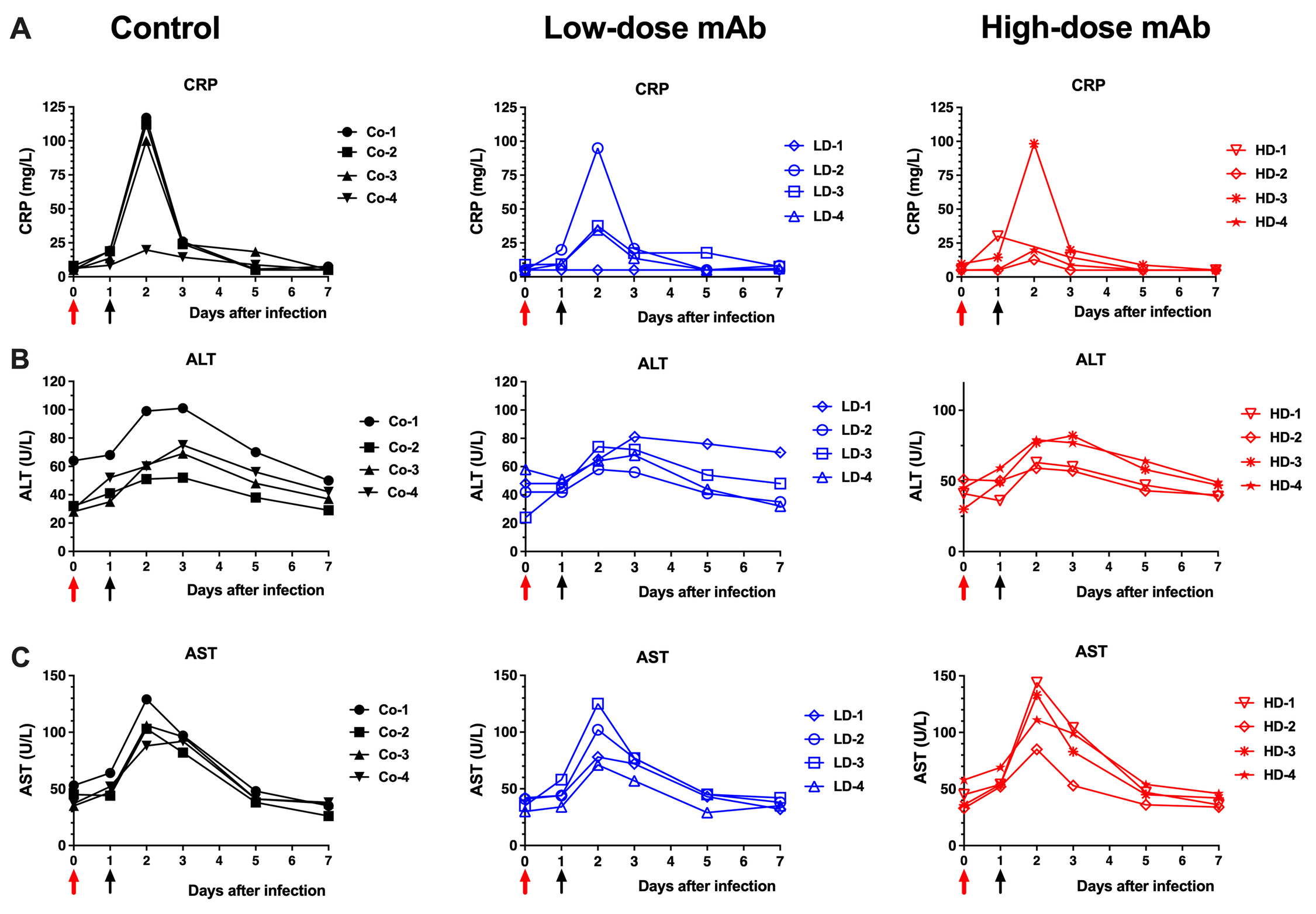

Supplement: S3 Fig — Biochemistry analysis on serum samples was performed using Piccolo® BioChemistry Plus disks. (A) through (C) present C-reactive protein (CRP), alanine aminotransferase (ALT), and aspartate aminotransferase (AST), which showed transient changes during the early stages of infection regardless of the study group. Other markers in the panel did not show any obvious changes. Red and black arrows indicate time of virus inoculation and monoclonal antibody administration on days 0 and 1, respectively. (TIF) [file ppat.1009688.s003.tif]

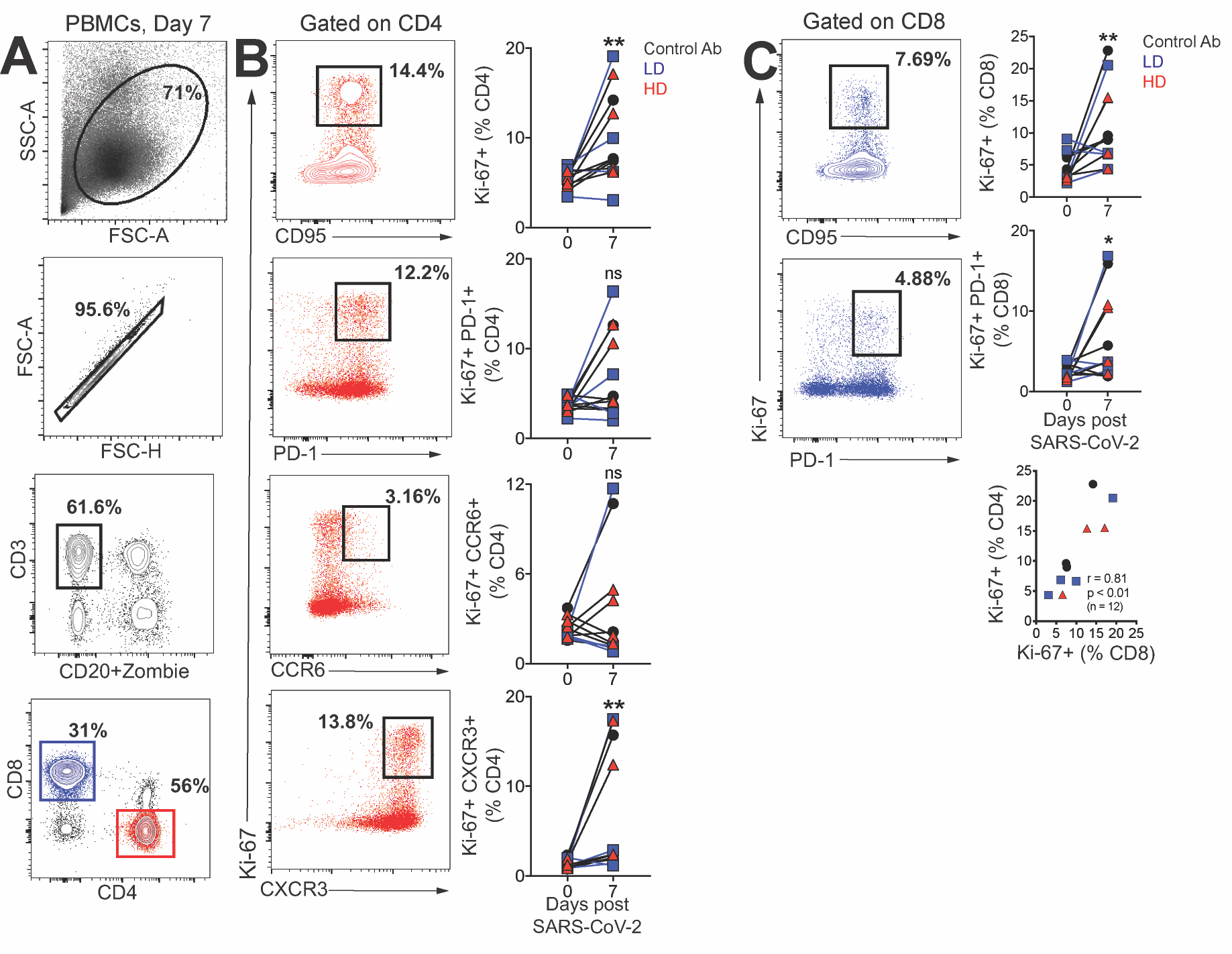

Supplement: S4 Fig — (A) Gating strategy to identify CD4 and CD8 T cells. (B) shows Ki-67+ CD4 T cells and expression of PD-1, CCR6, CXCR3 on proliferating CD4 T cells and respective kinetics. (C) shows induction of Ki-67+ CD8 T cells, expression of PD-1, and association between proliferating CD4 T and CD8 T cells. LD and HD indicate low-dose and high-dose mAb, respectively. (TIF) [file ppat.1009688.s004.tif]

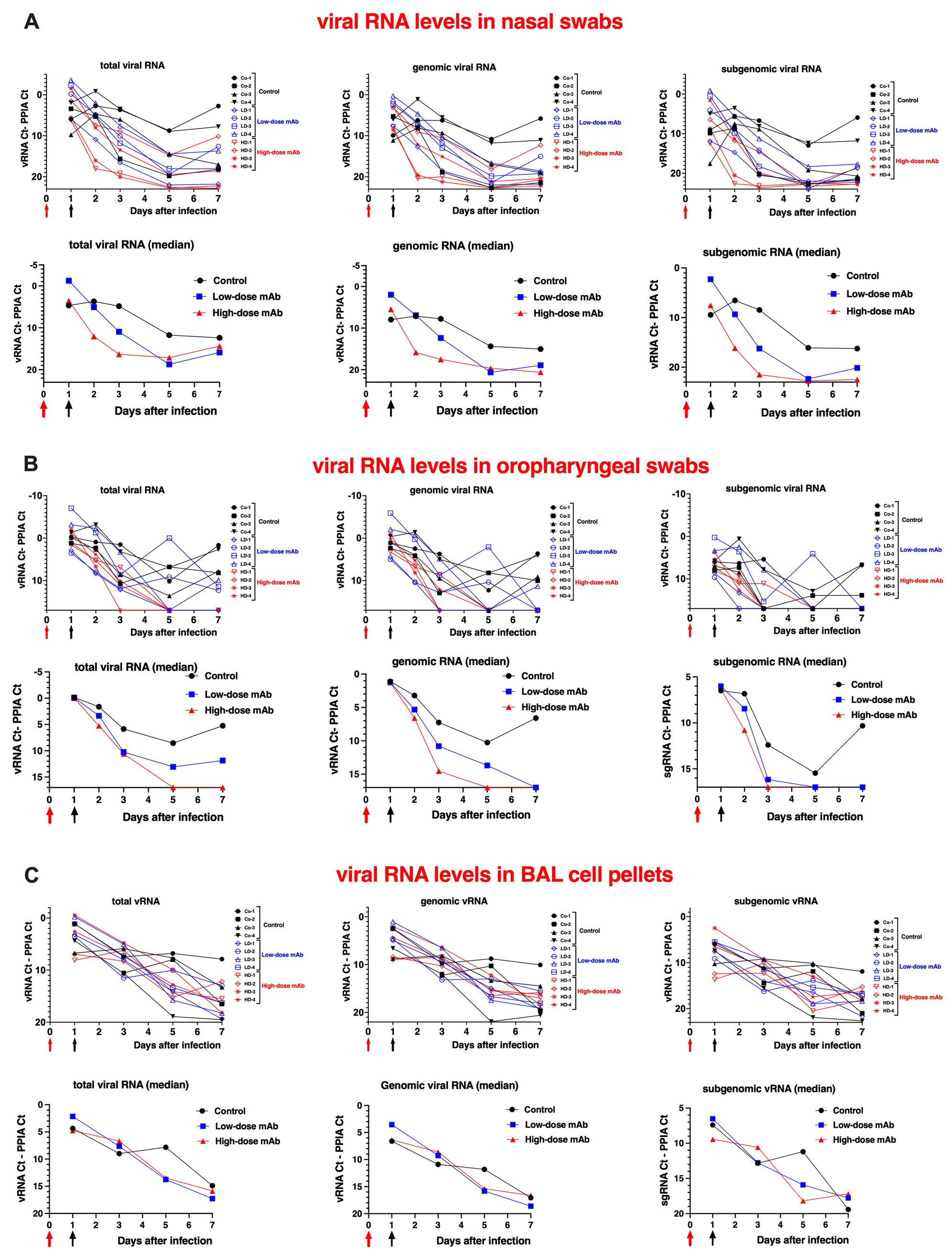

Supplement: S5 Fig — Nasal swabs (A), oropharyngeal swabs (B) and BAL cell pellets (C) were tested by RT-qPCR for total, genomic and subgenomic viral RNA, and the housekeeping gene PPIA mRNA. Viral RNA levels are expressed relative to PPIA mRNA by graphing the difference in Ct values. For each sample type, the top figures show the individual data (with the intersection of X-axis and Y-axis set near the limit of detection); the bottom figures display the median values per group. Red and black arrows indicate time of virus inoculation and monoclonal antibody administration on days 0 and 1, respectively. (TIF) [file ppat.1009688.s005.tif]

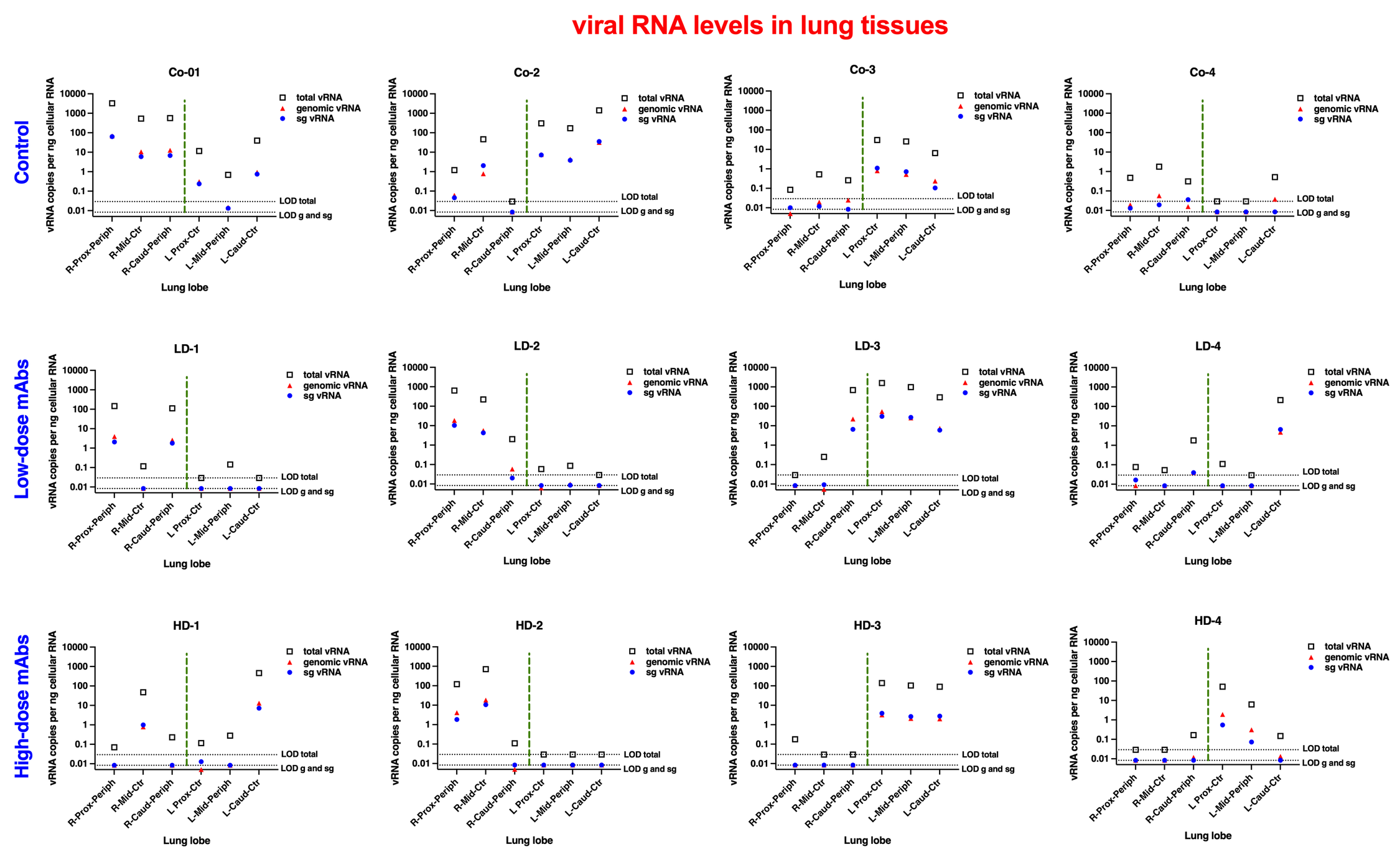

Supplement: S6 Fig — Specimens of 6 lung lobes were tested by RT-qPCR for total, genomic and subgenomic viral RNA (expressed relative to cellular RNA). For 3 lung lobes, only a peripheral section (removed before infusion of the rest of the lobe with formalin; see materials and methods) was available for viral load testing; for the other 3 lung lobes, a central specimen was tested for viral load. The pattern of detectable viral RNA reflected random dispersion among the lung lobes, with no discernible predilection of virus replication for left versus right lung lobes, or peripheral versus central lung tissue. The vertical line separates right from left side lung lobes. (TIF) [file ppat.1009688.s006.tif]

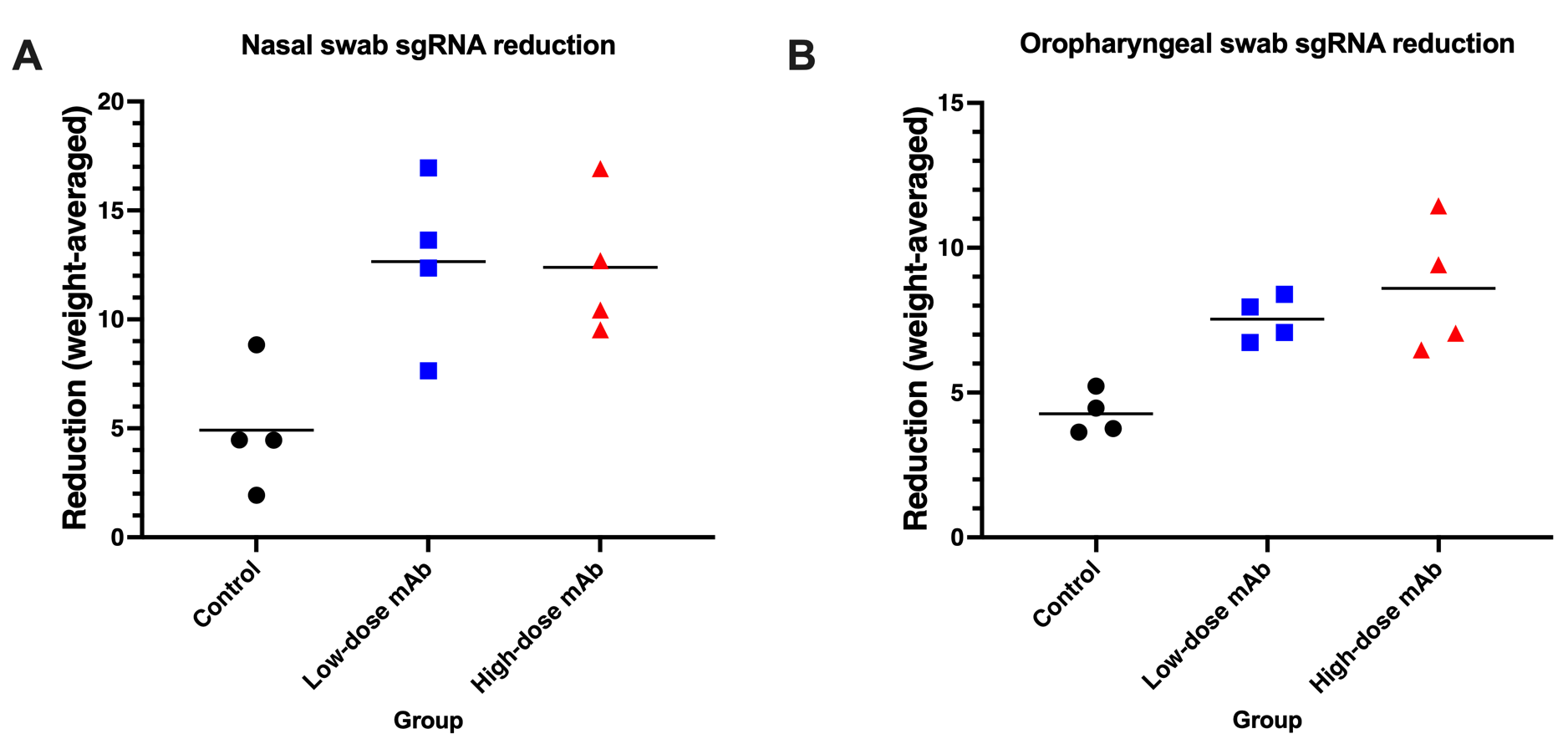

Supplement: S7 Fig — A weighted average analysis was performed on the sgRNA data in nasal and oropharyngeal swabs (S5 Fig) to calculate the relative decline of viral RNA (relative to cellular mRNA in the sample) from day 1 to day 7. For each animal, the AUC of relative sgRNA per cellular mRNA over time (S5 Fig) was tabulated using day 1 as baseline, and then divided by 6 days to get the weighted average in the decline of sgRNA over the 6-day time period. Lines indicate mean values. Statistical analysis revealed significant effects of monoclonal antibody treatment. For panel A, ANOVA: p = 0.016; Dunnett’s multiple comparison test: Control versus low-dose mAb: adjusted p = 0.018; Control versus high-dose mAb: adjusted p = 0.021. For panel B, ANOVA: p = 0.006; Dunnett’s multiple comparison test: control versus low-dose mAb: adjusted p = 0.02; control versus high-dose mAb: adjusted p = 0.004. (TIF) [file ppat.1009688.s007.tif]

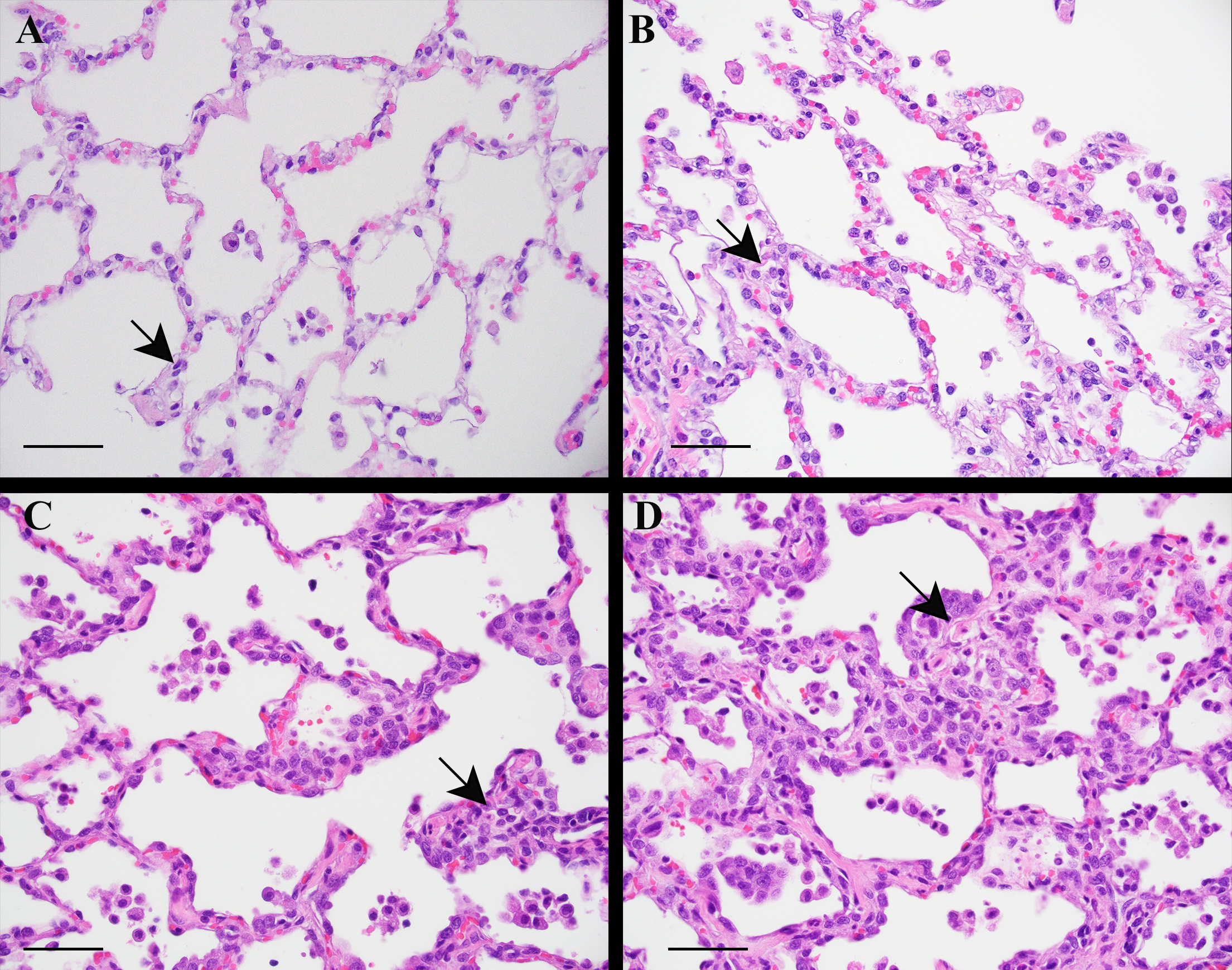

Supplement: S8 Fig — Interstitial cellularity score assigned to 25 unbiased random x40 fields per slide is based on the number of cells expanding the alveolar interstitium. Grade 1 (A) 1–2 cells thick, grade 2 (B) 3–4 cells thick, grade 3 (C) 5–6 cells thick, grade 4 (D) >6 cells thick. Bar = 50 μm. The score is allocated according to the most severe region within the field. Total score per animal is based on a weighted average of approximately 450 to 675 fields scored per animal. (TIF) [file ppat.1009688.s008.tif]

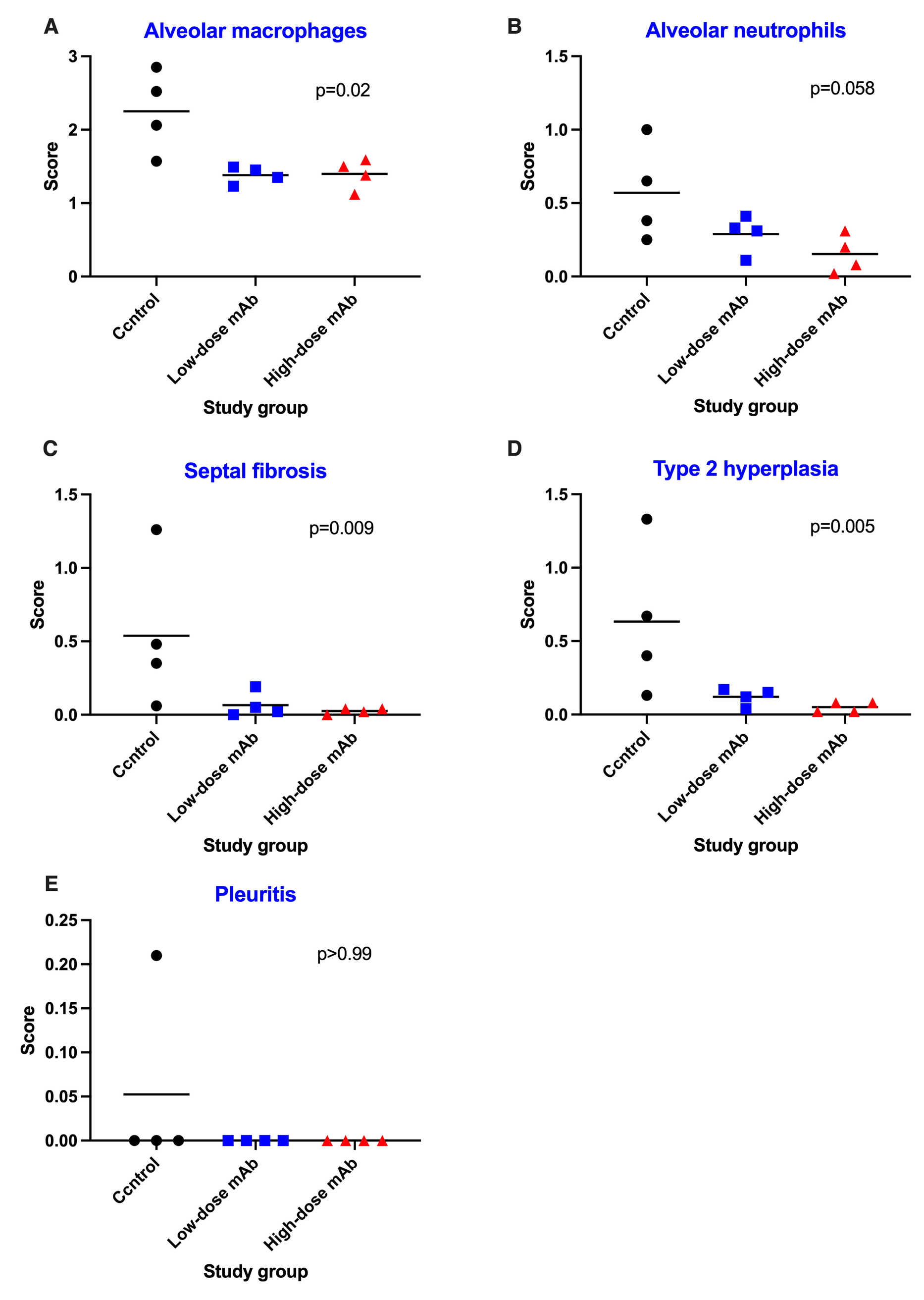

Supplement: S9 Fig — In addition to evaluation of lung septal cellularity as primary marker of interstitial pneumonia (see Fig 6), lung sections were also scored for other evidence of inflammation, injury and repair, including presence of alveolar macrophages (A), alveolar neutrophils (B), septal fibrosis (C), type 2 hyperplasia (D) and pleuritis (E) (as described in S6 Table). For each graph, p values represent Kruskal-Wallis test for comparison of the 3 study groups. C, LD and HD represent control, low-dose and high-dose SARS-CoV-2 mAb groups, respectively. For graph E, only one control animal (Co-3) had pleuritis. (TIF) [file ppat.1009688.s009.tif]
