# Supplementary material for: Early treatment with a combination of two potent neutralizing antibodies improves clinical outcomes and reduces virus replication and lung inflammation in SARS-CoV-2 infected macaques
Source: PLoS Pathog. 2021 Jul 6;17(7):e1009688. doi: 10.1371/journal.ppat.1009688 (PMC8284825; doi:10.1371/journal.ppat.1009688)
Supplement: S1 Table — Animals were inoculated with SARS-CoV-2 on day 0, and antibodies were infused on day 1. 50% and 90% neutralization titers (NT50 and NT90) in serum were measured by a RVPN assay. Concentrations of CoV-2 mAbs were calculated based on the NT50 and NT90 values of the RVPN curves for each sample and using the neutralization activity of the combination of antibodies. The mean values for the 2 CoV-2 mAb groups from day 2 to day 7 were calculated (bottom section) and reflect the ~3-fold differences in dosage between the 2 treatment groups. (DOCX) [file ppat.1009688.s010.docx]

**S1 Table**. **Neutralization titers and antibody concentrations in serum of macaques.**

Animals were inoculated with SARS-CoV-2 on day 0, and antibodies were infused on day 1. 50% and 90% neutralization titers (NT50 and NT90) in serum were measured by a RVPN assay. Concentrations of CoV-2 mAbs were calculated based on the NT_50_ and NT_90_ values of the RVPN curves for each sample and using the neutralization activity of the combination of antibodies. The mean values for the 2 CoV-2 mAb groups from day 2 to day 7 were calculated (bottom section) and reflect the ~3-fold differences in dosage between the 2 treatment groups.
