# Supplementary material for: Early treatment with a combination of two potent neutralizing antibodies improves clinical outcomes and reduces virus replication and lung inflammation in SARS-CoV-2 infected macaques
Source: PLoS Pathog. 2021 Jul 6;17(7):e1009688. doi: 10.1371/journal.ppat.1009688 (PMC8284825; doi:10.1371/journal.ppat.1009688)
Supplement: S2 Table — All thorax radiographs were scored blinded by a veterinary radiologist, with scores of 0 to 3 assigned to each of the 7 lung lobes. For each time point, the total score of all lung lobes was tabulated. (DOCX) [file ppat.1009688.s011.docx]

**S2 Table. Summary of radiological scoring.**


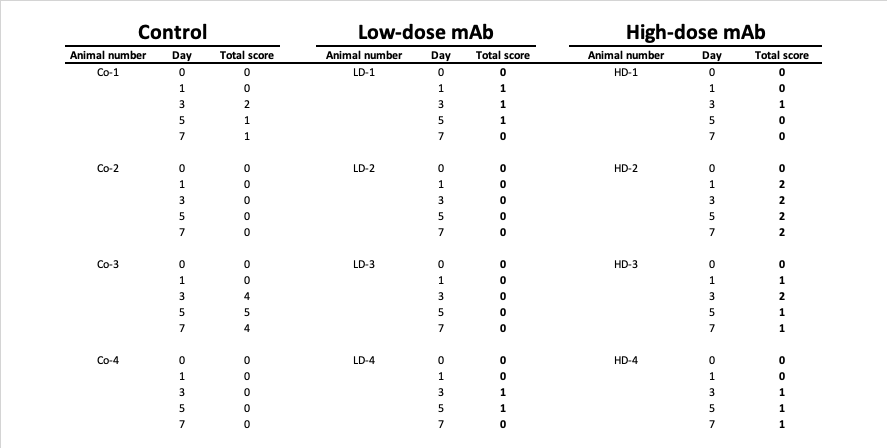


All thorax radiographs were scored blinded by a veterinary radiologist, with scores of 0 to 3 assigned to each of the 7 lung lobes. For each time point, the total score of all lung lobes was tabulated.
