# Supplementary material for: Early treatment with a combination of two potent neutralizing antibodies improves clinical outcomes and reduces virus replication and lung inflammation in SARS-CoV-2 infected macaques
Source: PLoS Pathog. 2021 Jul 6;17(7):e1009688. doi: 10.1371/journal.ppat.1009688 (PMC8284825; doi:10.1371/journal.ppat.1009688)
Supplement: S3 Table — The above table was adapted from WNPRC COVID scoring sheet (https://openresearch.labkey.com/wiki/Coven/page.view?name=clinical-scoring)), which itself was modified from a previous NHP influenza A virus study to include clinical signs relevant to COVID-19 and respiratory rates for cynomolgus macaques [1–3]. Cageside assessments were performed every day. The highest sum of scores for an animal determined the severity of disease. Clinical disease severity was classified as no clinical illness (0–4), mild (5–9), moderate (10–15), severe (>16). BPM = breaths per minute. 1. Chertow DS, Kindrachuk J, Sheng ZM, Pujanauski LM, Cooper K, Nogee D, Claire MS, Solomon J, Perry D, Sayre P, Janosko KB, Lackemeyer MG, Bohannon JK, Kash JC, Jahrling PB, Taubenberger JK. 2016. Influenza A and methicillin-resistant Staphylococcus aureus co-infection in rhesus macaques—A model of severe pneumonia. Antiviral Res. 129:120–129. doi:10.1016/j.antiviral.2016.02.013. 2. Bolton ID. 2015. Chapter 5—Basic Physiology of Macaca fascicularis. In The Nonhuman Primate in Nonclinical Drug Development and Safety Assessment. J Bluemel, S Korte, E Schenck, GF Weinbauer, editors. Academic Press, San Diego. 67–86. 3. Huang C, Wang Y, Li X, Ren L, Zhao J, Hu Y, Zhang L, Fan G, Xu J, Gu X, Cheng Z, Yu T, Xia J, Wei Y, Wu W, Xie X, Yin W, Li H, Liu M, Xiao Y, Gao H, Guo L, Xie J, Wang G, Jiang R, Gao Z, Jin Q, Wang J, Cao B. 2020. Clinical features of patients infected with 2019 novel coronavirus in Wuhan, China. Lancet. 395:497–506. doi:10.1016/S0140-6736(20)30183-5. (DOCX) [file ppat.1009688.s012.docx]

**S3 Table. Clinical Signs Scoring Criteria for SARS-CoV-2 infected macaques.**

| **Cage-Side Assessment** | | | |
| --- | --- | --- | --- |
| **Parameter** | **Description** | **Score** |  |
| Responsiveness and recumbency | Normal - bright, alert, responsive (note activity level, particularly if might affect RR) | 0 |  |
|  | Mild - slightly depressed, acts disinterested when personnel in room, lying down in cage but gets up when approached | 2 |  |
|  | Moderate/obtunded - non-responsive, very disinterested in personnel, hunched or lying down, will get up when prodded, pinched, or similarly stimulated | 4 |  |
|  | Severe/comatose - lying down completely unresponsive to stimuli | 6 |  |
| Discharges | Nasal or ocular not present during observation window, noted later (unrelated to procedures) | 1 |  |
|  | Mild nasal or ocular | 2 |  |
|  | Severe nasal or ocular | 4 |  |
| Skin | Normal | 0 |  |
|  | Mild dermatitis | 1 |  |
|  | Severe dermatitis | 3 |  |
| Respiration, dyspnea, and cough | Normal - no apparent changes in breathing, 30-54 BPM, and no cough | 0 |  |
|  | Very Mild – no changes in breathing, infrequent coughing or sneezing outside of observation window (unrelated to procedures) | 1 |  |
|  | Mild - slightly increased effort breathing, 55-65 BPM, or mild cough or sneezing | 2 |  |
|  | Moderate - obvious difficulty breathing, 66-80 BPM, or apparent cough | 4 |  |
|  | Severe - respirations labored, open mouth breathing, abdominal breathing, >80 BPM, cyanosis, or haemoptysis | 6 |  |
| Food consumption | <1/3 of chow and supplements remaining | 0 |  |
|  | 1/3-2/3 of chow and supplements remaining | 1 |  |
|  | >2/3 of chow and supplements remaining | 2 |  |
|  | Anorexia | 3 |  |
| Fecal consistency | Normal | 0 |  |
|  | Soft | 1 |  |
|  | Fluid | 2 |  |
|  | Fluid and profuse amount | 3 |  |
| **Total** | | |  |
| **Notes** *(any observed sneezing, vomit, conjunctival erythema, or other abnormalities*) | | | |

The above table was adapted from WNPRC COVID scoring sheet (<https://openresearch.labkey.com/wiki/Coven/page.view?name=clinical-scoring)>, which itself was modified from a previous NHP influenza A virus study to include clinical signs relevant to COVID-19 and respiratory rates for cynomolgus macaques [1–3]. Cageside assessments were performed every day. The highest sum of scores for an animal determined the severity of disease. Clinical disease severity was classified as no clinical illness (0-4), mild (5-9), moderate (10-15), severe (>16). BPM = breaths per minute.

1. Chertow DS, Kindrachuk J, Sheng ZM, Pujanauski LM, Cooper K, Nogee D, Claire MS, Solomon J, Perry D, Sayre P, Janosko KB, Lackemeyer MG, Bohannon JK, Kash JC, Jahrling PB, Taubenberger JK. 2016. Influenza A and methicillin-resistant Staphylococcus aureus co-infection in rhesus macaques - A model of severe pneumonia. Antiviral Res. 129:120-129. doi:10.1016/j.antiviral.2016.02.013.
2. Bolton ID. 2015. Chapter 5 - Basic Physiology of Macaca fascicularis. In The Nonhuman Primate in Nonclinical Drug Development and Safety Assessment. J Bluemel, S Korte, E Schenck, GF Weinbauer, editors. Academic Press, San Diego. 67-86.
3. Huang C, Wang Y, Li X, Ren L, Zhao J, Hu Y, Zhang L, Fan G, Xu J, Gu X, Cheng Z, Yu T, Xia J, Wei Y, Wu W, Xie X, Yin W, Li H, Liu M, Xiao Y, Gao H, Guo L, Xie J, Wang G, Jiang R, Gao Z, Jin Q, Wang J, Cao B. 2020. Clinical features of patients infected with 2019 novel coronavirus in Wuhan, China. Lancet. 395:497-506. doi:10.1016/S0140-6736(20)30183-5.
