# Supplementary material for: Early treatment with a combination of two potent neutralizing antibodies improves clinical outcomes and reduces virus replication and lung inflammation in SARS-CoV-2 infected macaques
Source: PLoS Pathog. 2021 Jul 6;17(7):e1009688. doi: 10.1371/journal.ppat.1009688 (PMC8284825; doi:10.1371/journal.ppat.1009688)
Supplement: S4 Table — The above table was adapted from WNPRC COVID scoring sheet (https://openresearch.labkey.com/wiki/Coven/page.view?name=clinical-scoring), which itself was modified from a previous NHP influenza A virus study to include clinical signs relevant to COVID-19 and respiratory rates for cynomolgus macaques [1–3]. Physical examinations were performed whenever an animal was anesthetized. The highest sum of scores for an animal determined the severity of disease. Clinical disease severity was classified as no clinical illness (0–2), mild (3–7), moderate (8–13), severe (>13). BPM = breaths per minute for respiratory rate and beats per minute for heart rate. 1. Chertow DS, Kindrachuk J, Sheng ZM, Pujanauski LM, Cooper K, Nogee D, Claire MS, Solomon J, Perry D, Sayre P, Janosko KB, Lackemeyer MG, Bohannon JK, Kash JC, Jahrling PB, Taubenberger JK. 2016. Influenza A and methicillin-resistant Staphylococcus aureus co-infection in rhesus macaques—A model of severe pneumonia. Antiviral Res. 129:120–129. doi:10.1016/j.antiviral.2016.02.013. 2. Bolton ID. 2015. Chapter 5—Basic Physiology of Macaca fascicularis. In The Nonhuman Primate in Nonclinical Drug Development and Safety Assessment. J Bluemel, S Korte, E Schenck, GF Weinbauer, editors. Academic Press, San Diego. 67–86. 3. Huang C, Wang Y, Li X, Ren L, Zhao J, Hu Y, Zhang L, Fan G, Xu J, Gu X, Cheng Z, Yu T, Xia J, Wei Y, Wu W, Xie X, Yin W, Li H, Liu M, Xiao Y, Gao H, Guo L, Xie J, Wang G, Jiang R, Gao Z, Jin Q, Wang J, Cao B. 2020. Clinical features of patients infected with 2019 novel coronavirus in Wuhan, China. Lancet. 395:497–506. doi:10.1016/S0140-6736(20)30183-5. (DOCX) [file ppat.1009688.s013.docx]

**S4 Table. Physical examination under anesthesia scoring criteria.**

| **Physical Examination Under Anesthesia** | | | |
| --- | --- | --- | --- |
| **Parameter** | **Description** | **Score** |  |
| Rectal temperature *(taken immediately after sedation)* | Normal (100.0-102.4F) | 0 |  |
|  | Mild Hypothermia (98.0-99.9F) or Hyperthermia (102.5-103.4F) | 2 |  |
|  | Moderate Hypothermia (96.0-97.9F) or Hyperthermia (103.5-104.4F) | 4 |  |
|  | Severe hypothermia (<96.0) or Severe hyperthermia (>104.4F) | 6 |  |
| Heart rate and blood pressure | Normal (<160 BPM, Baseline BP +/- 15%) | 0 |  |
|  | Mild tachycardia (160-<180 BPM), normotensive | 1 |  |
|  | Moderate tachycardia (180-<200 BPM), normotensive | 2 |  |
|  | Severe tachycardia (>200 BPM), hypotensive (<85% of baseline) | 3 |  |
| Respiratory rate | Normal - 30-54 BPM | 0 |  |
|  | Mild tachypnea- 55-65 BPM | 2 |  |
|  | Moderate tachypnea - 66-80 BPM | 4 |  |
|  | Severe tachypnea - >80 BPM | 6 |  |
| Respiratory character | Normal | 0 |  |
|  | Mild dyspnea | 1 |  |
|  | Severe dyspnea | 3 |  |
| SpO_2_ | Normal (95-100%) | 0 |  |
|  | Mildly decreased (90-94%) | 1 |  |
|  | Moderately decreased (87-89) | 2 |  |
|  | Severely decreased (<87) | 3 |  |
| Body weight | Normal (0-3% loss) | 0 |  |
|  | Mild (4-9% loss) | 1 |  |
|  | Moderate (10-16% loss) | 2 |  |
|  | Severe (>16% loss) | 3 |  |
| Hydration | Normal skin turgor, moist mucous membranes | 0 |  |
|  | Skin tenting, greater than 2 sec return | 1 |  |
|  | Skin tenting with incomplete return | 2 |  |
|  | Skin tenting prolonged and sunken eyes | 3 |  |
| **Total** | | |  |
| **Notes** *(auscultation findings if applicable, conjunctival erythema, palpable masses, or any other abnormalities)* | | | |

The above table was adapted **from WNPRC COVID scoring sheet (**[**https://openresearch.labkey.com/wiki/Coven/page.view?name=clinical-scoring**](https://openresearch.labkey.com/wiki/Coven/page.view?name=clinical-scoring)**),** which itself was modified from a previous NHP influenza A virus study to include clinical signs relevant to COVID-19 and respiratory rates for cynomolgus macaques [1–3]. Physical examinations were performed whenever an animal was anesthetized. The highest sum of scores for an animal determined the severity of disease. Clinical disease severity was classified as no clinical illness (0-2), mild (3-7), moderate (8-13), severe (>13). BPM = breaths per minute for respiratory rate and beats per minute for heart rate.

1. Chertow DS, Kindrachuk J, Sheng ZM, Pujanauski LM, Cooper K, Nogee D, Claire MS, Solomon J, Perry D, Sayre P, Janosko KB, Lackemeyer MG, Bohannon JK, Kash JC, Jahrling PB, Taubenberger JK. 2016. Influenza A and methicillin-resistant Staphylococcus aureus co-infection in rhesus macaques - A model of severe pneumonia. Antiviral Res. 129:120-129. doi:10.1016/j.antiviral.2016.02.013.
2. Bolton ID. 2015. Chapter 5 - Basic Physiology of Macaca fascicularis. In The Nonhuman Primate in Nonclinical Drug Development and Safety Assessment. J Bluemel, S Korte, E Schenck, GF Weinbauer, editors. Academic Press, San Diego. 67-86.
3. Huang C, Wang Y, Li X, Ren L, Zhao J, Hu Y, Zhang L, Fan G, Xu J, Gu X, Cheng Z, Yu T, Xia J, Wei Y, Wu W, Xie X, Yin W, Li H, Liu M, Xiao Y, Gao H, Guo L, Xie J, Wang G, Jiang R, Gao Z, Jin Q, Wang J, Cao B. 2020. Clinical features of patients infected with 2019 novel coronavirus in Wuhan, China. Lancet. 395:497-506. doi:10.1016/S0140-6736(20)30183-5.
