# Supplementary material for: Early treatment with a combination of two potent neutralizing antibodies improves clinical outcomes and reduces virus replication and lung inflammation in SARS-CoV-2 infected macaques
Source: PLoS Pathog. 2021 Jul 6;17(7):e1009688. doi: 10.1371/journal.ppat.1009688 (PMC8284825; doi:10.1371/journal.ppat.1009688)
Supplement: S5 Table — (DOCX) [file ppat.1009688.s014.docx]

**S5 Table. Flow cytometry antibody and reagents.**

| **S.No** | **Reagents** | **Source** | **Identifier** |
| --- | --- | --- | --- |
| 1. | FITC anti-human MPO (clone 5B8) | BD Biosciences | Cat#340580 |
| 2. | AF700 anti-human CD14 (clone MSE2) | BD Biosciences | Cat# 301822 |
| 3. | APC anti-human CD66 (clone TET2) | Miltenyi Biotec | Order#130-118-539 |
| 4. | APC-Cy7 anti-human CD3 (clone SP34-2) | BD Biosciences | Cat#557757 |
| 5. | APC-Cy7 anti-human CD20 (clone 2H7) | BioLegend | Cat#302314 |
| 6. | APC-Cy7 anti-human live/dead | invitrogen | Ref#L34976A |
| 7. | PECy7 anti-human CD11c (clone 3.9) | invitrogen | Ref#25-0116-42 |
| 8. | PECF594 anti-human CX3CR1 (clone 2A9-1) | BioLegend | Cat#341624 |
| 9. | PE anti-human CD163 (clone GHI/61) | BioLegend | Cat#333606 |
| 10. | BV605 anti-human CD16 (clone 3G8) | BioLegend | Cat#302040 |
| 11. | BV650 anti-human CCR7 (clone 3D12) | BD Biosciences | Cat#563407 |
| 12. | BV786 anti-human HLA-DR (clone L243) | BioLegend | Cat#307642 |
| 13. | BV510 anti-human CD11b (clone ICRF44) | BioLegend | Cat#301334 |
| 14. | BV421 anti-human CD123 (clone 7G3) | invitrogen | Ref#48-1238-42 |
| 15. | BUV805 anti-human CD8 (clone SK1) | BD Biosciences | Cat#612889 |
| 16 | FACS lyse | BD Biosciences | Cat#349202 |
| 17 | Cytofix/Cytoperm | BD Biosciences | Cat#51-2090KZ |
| 18 | Brilliant stain buffer | BD Biosciences | Cat#563794 |
| 19 | Perm/wash buffer | BD Biosciences | Cat#554723 |
