# Supplementary material for: Early treatment with a combination of two potent neutralizing antibodies improves clinical outcomes and reduces virus replication and lung inflammation in SARS-CoV-2 infected macaques
Source: PLoS Pathog. 2021 Jul 6;17(7):e1009688. doi: 10.1371/journal.ppat.1009688 (PMC8284825; doi:10.1371/journal.ppat.1009688)
Supplement: S6 Table — A single score per slide was given based on the criteria provided above. Total score per animal was provided as a weighted average. (DOCX) [file ppat.1009688.s015.docx]

**S6 Table. Additional scoring system of lung histology.**

| **Histology finding** | **0** | **1** | **2** | **3** | **4** |
| --- | --- | --- | --- | --- | --- |
| Alveolar macrophages | 0-5/slide | Rare; 1-3 cells in at least one x40 field | Hard to find; 5-10 individual cells in at least in one x40 field; no clusters | Easy to find; 10-20 cells in at least one x40 field; rare clusters | 10-20 cells in majority of x40 fields; clusters are common |
| Alveolar neutrophils | Absent | Rare; presence of any neutrophils scored at least a 1 | Hard to find; 5-10 individual cells anywhere on the slide | Easy to find; 10-20 cells in a x40 field | Prominent finding; more than 20 cells in multiple x40 fields |
| Type II hyperplasia | Absent | Present; Less than 10% of lesion | More than 10% of the lesion |  |  |
| Septal Fibrosis | Absent | Present; Less than 10% of lesion | More than 10% of the lesion |  |  |
| Pleuritis | Absent | Present |  |  |  |

A single score per slide was given based on the criteria provided above. Total score per animal was provided as a weighted average.
